# RsaL is a self‐regulatory switch that controls alternative biosynthesis of two AHL‐type quorum sensing signals in *Pseudomonas aeruginosa* PA1201

**DOI:** 10.1002/mlf2.12113

**Published:** 2024-03-18

**Authors:** Ya‐Wen He, Zi‐Jing Jin, Ying Cui, Kai Song, Bo Chen, Lian Zhou

**Affiliations:** ^1^ State Key Laboratory of Microbial Metabolism, Joint International Research Laboratory of Metabolic and Developmental Sciences, SJTU‐NLBP Joint R&D Centre for Biopesticides and Biofertilizers, School of Life Sciences and Biotechnology Shanghai Jiao Tong University Shanghai China; ^2^ Zhiyuan Innovative Research Centre, Student Innovation Centre, Zhiyuan College Shanghai Jiao Tong University Shanghai China

**Keywords:** *N*‐3‐oxo‐dodecanoyl homoserine lactone, *N*‐butanoyl‐homoserine lactone, *Pseudomonas aeruginosa*, quorum sensing, RsaL

## Abstract

*Pseudomonas aeruginosa* is a ubiquitous and metabolically versatile microorganism naturally found in soil and water. It is also an opportunistic pathogen in plants, insects, animals, and humans. In response to increasing cell density, *P*. *aeruginosa* uses two acyl‐homoserine lactone (AHL) quorum‐sensing (QS) signals (i.e., *N*‐3‐oxo‐dodecanoyl homoserine lactone [3‐oxo‐C12‐HSL] and *N*‐butanoyl‐homoserine lactone [C4‐HSL]), which regulate the expression of hundreds of genes. However, how the biosynthesis of these two QS signals is coordinated remains unknown. We studied the regulation of these two QS signals in the rhizosphere strain PA1201. PA1201 sequentially produced 3‐oxo‐C12‐HSL and C4‐HSL at the early and late growth stages, respectively. The highest 3‐oxo‐C12‐HSL‐dependent elastase activity was observed at the early stage, while the highest C4‐HSL‐dependent rhamnolipid production was observed at the late stage. The atypical regulator RsaL played a pivotal role in coordinating 3‐oxo‐C12‐HSL and C4‐HSL biosynthesis and QS‐associated virulence. RsaL repressed *lasI* transcription by binding the –10 and –35 boxes of the *lasI* promoter. In contrast, RsaL activated *rhlI* transcription by binding the region encoding the 5′‐untranslated region of the *rhlI* mRNA. Further, RsaL repressed its own expression by binding a nucleotide motif located in the –35 box of the *rsaL* promoter. Thus, RsaL acts as a molecular switch that coordinates the sequential biosynthesis of AHL QS signals and differential virulence in PA1201. Finally, C4‐HSL activation by RsaL was independent of the Las and *Pseudomonas* quinolone signal (PQS) QS signaling systems. Therefore, we propose a new model of the QS regulatory network in PA1201, in which RsaL represents a superior player acting at the top of the hierarchy.

## Impact statement

Quorum sensing (QS) regulation allows bacteria to control their gene expression in response to their population density. *Pseudomonas aeruginosa* strain PA1201 uses two acyl‐homoserine lactone (AHL) QS signals, *N*‐3‐oxo‐dodecanoyl homoserine lactone (3‐oxo‐C12‐HSL) and *N*‐butanoyl‐homoserine lactone (C4‐HSL), to regulate gene expression. It produces sequentially 3‐oxo‐C12‐HSL and C4‐HSL at early and late growth stages, respectively. RsaL is a self‐induced regulatory protein. It acts as a molecular switch to regulate the alternative biosynthesis of 3‐oxo‐C12‐HSL and C4‐HSL via differentially binding to the promoter regions of *lasI* and *rhlI*. Thus, RsaL represents a superior player acting at the top of the hierarchy that coordinates sequential QS signal biosynthesis for cell density‐dependent gene expression.

## INTRODUCTION

Many bacteria use chemical signals to communicate between cells in a process called quorum sensing (QS). QS allows bacteria to sense population density and coordinate their behavior through gene regulation^1^. *Pseudomonas aeruginosa* is a ubiquitous and metabolically versatile microorganism naturally found in soil and water. However, it is also an opportunistic pathogen in plants and animals, including insects and humans^2^, ^3^, ^4^. As a reflection of its versatile tropism and metabolism, *P. aeruginosa* possesses a complex QS network. The acyl‐homoserine lactone (AHL)‐ and quinolone‐dependent QS (*Pseudomonas* quinolone signal [PQS]) systems have been extensively studied in the model strain PAO1, revealing a hierarchical network that regulates hundreds of genes in response to increasing cell density^5^. In AHL‐dependent systems, two acyl‐homoserine lactone (HSL) synthase enzymes, LasI and RhlI, are responsible for the biosynthesis of the QS signals *N*‐3‐oxo‐dodecanoyl homoserine lactone (3‐oxo‐C12‐HSL) and *N*‐butanoyl‐homoserine lactone (C4‐HSL), respectively. 3‐oxo‐C12‐HSL and C4‐HSL bind their cognate transcriptional factors LasR and RhlR to regulate the expression of downstream target genes^5^, ^6^. Interestingly, many genes are exclusively regulated by one of the two acyl‐HSLs, while others respond to both in the model strain PAO1^7^. In addition, QS activity in PAO1 is modulated by a number of regulatory factors, including Vfr, QteE, CdpR, the LuxR homologs QscR and VqsR, and RsaL, constituting a complex hierarchical regulatory network^5^, ^8^, ^9^, ^10^.

Intriguingly, when PAO1 is cultured in complex media, the 3‐oxo‐C12‐HSL concentration plateaus during the late logarithmic growth phase, while the C4‐HSL level increases continuously^8^, ^11^. *P. aeruginosa* PA1201 was originally identified as a biocontrol strain in the rice rhizosphere^12^. Unlike the clinically isolated strain PAO1, PA1201 is less toxic to both human cell lines and *Drosophila melanogaster* ^13^. The PA1201‐derived strain UP46 has been used to industrially produce the biopesticide Shenqinmycin in China^13^. Our previous results on PA1201 confirmed a high level of C4‐HSL in the late logarithmic growth phase^14^. However, how and why *P. aeruginosa* differentially regulates the production of two types of AHL QS signals, depending on its growth phase and specific environments, remain to be understood.

In PAO1, the small regulatory protein RsaL maintains 3‐oxo‐C12‐HSL homeostasis. *rsaL* lies in the intergenic region between *lasR* and *lasI* and encodes a transcriptional regulator belonging to a subfamily of the tetrahelical superclass of helix‐turn‐helix proteins^8^, ^15^, ^16^. *rsaL* and *lasI* share an overlapping bidirectional promoter. RsaL represses *lasI* expression at high cell densities, which antagonizes LasR activity and limits 3‐oxo‐C12‐HSL production to a physiological concentration in PAO1^8^. DNase I footprint analysis has identified a conserved RsaL‐binding sequence (TATGnAAnTTnCATA) overlapping the –10 box of the *lasI* promoter (P_lasI_)^8^. However, whether and how RsaL regulates the biosynthesis of another AHL‐type QS signal, C4‐HSL, remain unclear in *P*. *aeruginosa*.

Besides its role in QS, RsaL is also a global regulator controlling the expression of numerous genes involved in bacterial functions such as virulence, including the *phz1* cluster, *phzM*, and *cdpR*, and in antibiotic resistance, including the *narK1K2GHJI* operon^8^, ^14^, ^17^, ^18^, ^19^. Consequently, the *rsaL* mutation in the *P*. *aeruginosa* strain PAO1 enhances the production of virulence factors, such as elastase, hemolysins, hydrogen cyanide, and phenazine metabolites, increases ciprofloxacin and carbenicillin resistance and decreases the ability of the bacteria to cause chronic lung infections in mice^15^, ^19^, ^20^, ^21^, ^22^. Therefore, understanding the RsaL‐dependent regulation of gene expression may uncover new ways to control the harmful effects of *P*. *aeruginosa* in clinics and agriculture.

In this study, the rhizosphere *P*. *aeruginosa* strain PA1201 was used as a model to decipher the regulation of the two AHL QS signals, 3‐oxo‐C12‐HSL and C4‐HSL, and their effects on elastase and rhamnolipid biosynthesis during bacterial growth. We demonstrated that RsaL plays an essential role in the regulation of these alternative pathways and investigated the molecular mechanisms whereby RsaL represses its own and *lasI* expression and activates *rhlI* expression. These findings led to a new model in which RsaL acts as a molecular switch that coordinates alternative AHL QS signals in *P*. *aeruginosa*, which in turn triggers differential gene expression programs in response to cell density and environmental changes.

## RESULTS

### 3‐oxo‐C12‐HSL and C4‐HSL biosynthesis alternatives during PA1201 growth

**Figure 1:**
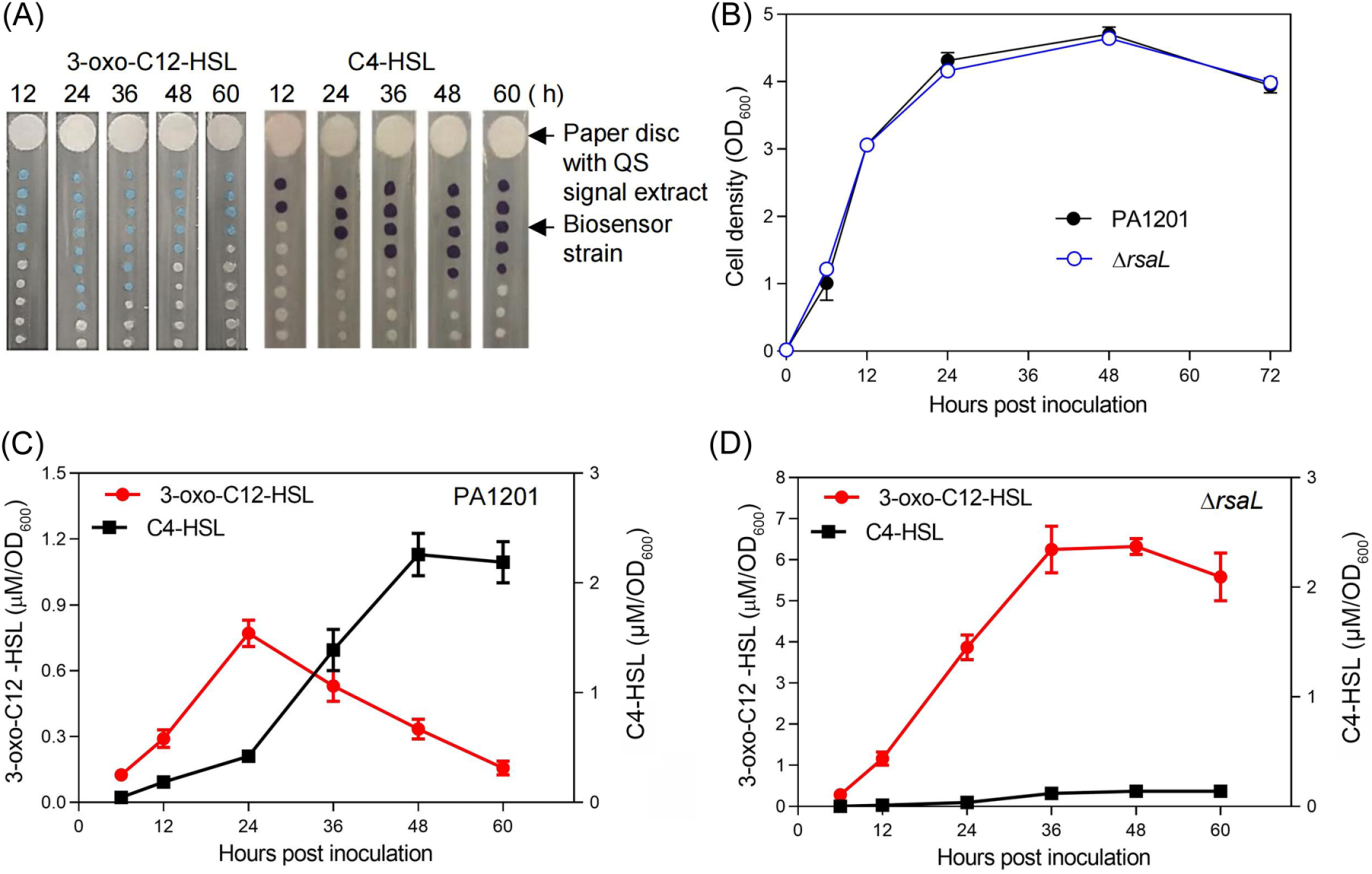
Kinetics of 3‐oxo‐C12‐HSL and C4‐HSL production by PA1201 and Δ*rsaL* grown in PPM liquid medium. (A) 3‐oxo‐C12‐HSL and C4‐HSL relative levels evaluated by their respective biosensor strains, CF11 and CV026. (B) Growth curves of PA1201 and Δ*rsaL* cultured in PPM medium. (C, D) Quantification of 3‐oxo‐C12‐HSL (left *y‐*axis) and C4‐HSL (right *y‐*axis) levels in PA1201 (C) and Δ*rsaL* (D) cultures by UPLC‐MS. Three independent experiments were conducted; averages and standard deviations are shown. 3‐oxo‐C12‐HSL, *N*‐3‐oxo‐dodecanoyl homoserine lactone; C4‐HSL, *N*‐butanoyl‐homoserine lactone; PPM, pigment‐promoting medium; UPLC‐MS, ultra‐high performance liquid chromatography‐mass spectrometry.

*P. aeruginosa* produces two types of AHL‐type QS signals: 3‐oxo‐C12‐HSL and C4‐HSL^5^. 3‐oxo‐C12‐HSL and C4‐HSL molecules produced by PA1201 in pigment‐promoting medium (PPM) culture were extracted, and their relative levels were evaluated using the biosensor strains *Agrobacterium tumefaciens* CF11 and *Chromobacterium violaceum* CV026, respectively^23^. 3‐oxo‐C12‐HSL peaked at 24 hours postinoculation (hpi) and declined over time (Figure 1A). In contrast, the C4‐HSL level increased progressively during growth and reached a maximum plateau at 48 hpi that persisted until 60 hpi (Figure 1A).

The levels of 3‐oxo‐C12‐HSL and C4‐HSL produced by PA1201 in PPM were further quantified using a previously described ultra‐high performance liquid chromatography‐mass spectrometry (UPLC‐MS) method^14^. This quantification confirmed that the 3‐oxo‐C12‐HSL level reached a maximum at 24 hpi (0.77 μM/OD_600_) and then declined progressively to 0.15 μM/OD_600_ at 60 hpi (Figure 1C). In contrast, the C4‐HSL level remained low at the early growth stage (i.e., between 12 and 24 hpi), increased rapidly from 36 hpi onwards, and peaked at 2.25 μM/OD_600_ at 48 hpi (Figure 1C). These results suggest that PA1201 coordinates 3‐oxo‐C12‐HSL and C4‐HSL biosynthesis during growth and that this differential production operates a transcriptional switch during growth.

### 3‐oxo‐C12‐HSL‐ and C4‐HSL‐dependent virulence factors are alternatively induced during growth

**Figure 2:**
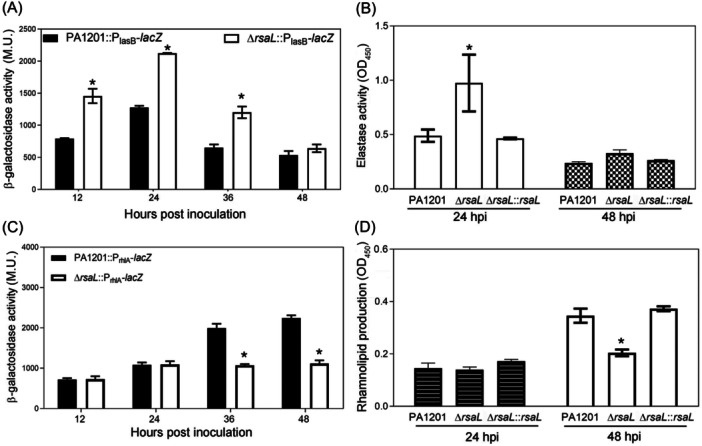
Monitoring of *lasB* and *rhlA* expression and corresponding elastase activity and rhamnolipid production in PA1201‐derived strains. (A) Relative transcriptional activity of the *LacZ* reporter gene under the control of the *lasB* promoter in the strains PA1201::P_lasB_‐*lacZ* and Δ*rsaL*:: P_lasB_‐*lacZ* in Miller units (M.U.) at different growth time points. (B) Elastase activity in the strains PA1201, Δ*rsaL*, and Δ*rsaL*::*rsaL* at 24 and 48 hours postinoculation (hpi). (C) Relative transcriptional activity of the *LacZ* reporter gene under the control of the *rhlA* promoter in the strains PA1201::P_rhlA_‐*lacZ* and Δ*rsaL*::P_rhlA_‐*lacZ* at different growth time points. (D) Rhamnolipid levels in the strains PA1201, Δ*rsaL*, and Δ*rsaL*::*rsaL* at 24 and 48 hpi. Three independent experiments were conducted; averages and standard deviations are shown. Statistically significant differences are indicated by one asterisk (*p* < 0.05). M.U., Miller units.

To test whether PA1201 can alternatively regulate gene expression through the control of 3‐oxo‐C12‐HSL and C4‐HSL biosynthesis, QS‐regulated gene expression and virulence factor production were monitored during growth. In *P. aeruginosa*, *lasB*, which encodes the predominant protease elastase, is one of the main target genes activated by the LasI/LasR system^24^. We cloned the *lasB* promoter (P_lasB_) and generated a *lacZ* fusion reporter strain, PA1201::P_lasB_‐*lacZ*, to monitor *lasB* expression. This experiment showed that P_lasB_ activity increased from 790 Miller units (M.U.) at 12 hpi to 1277.5 M.U. at 24 hpi and then decreased to 650 M.U. at 36 hpi (Figure 2A). Consistently, in the PA1201 culture, *lasB*‐dependent elastase activity was significantly higher at 24 hpi (0.49 OD_450_) than that at 48 hpi (0.24 OD_450_) (Figure 2B). Thus, during PA1201 growth, the peaks of *lasB* expression and elastase activity coincided with the highest level of 3‐oxo‐C12‐HSL.

The *rhlAB* operon encoding rhamnosyltransferase 1, an enzyme involved in the synthesis of the surfactant mono‐rhamnolipid, is activated by the RhlI/RhlR system^25^. We generated the reporter strain PA1201::P_rhlA_‐*lacZ* by cloning the promoter of *rhlA* (P_rhlA_) upstream of the *lacZ* reporter gene to monitor *rhlA* expression. The peak of P_rhlA_ activity was observed at 36 and 48 hpi (Figure 2C). Similarly, the relative rhamnolipid level at 24 hpi (0.14 OD_450_) was significantly lower than that at 48 hpi (0.34 OD_450_) (Figure 2D). Thus, the kinetics of *rhlA* expression and rhamnolipid production mirrored that of C4‐HSL. Taken together, these results suggest that PA1201 coordinates the production of elastase at the early growth stage and of rhamnolipid at the late growth stage during growth.

### RsaL is required for switching between the two HSL‐dependent QS signals and between elastase versus rhamnolipid production in PA1201

**Figure 3:**
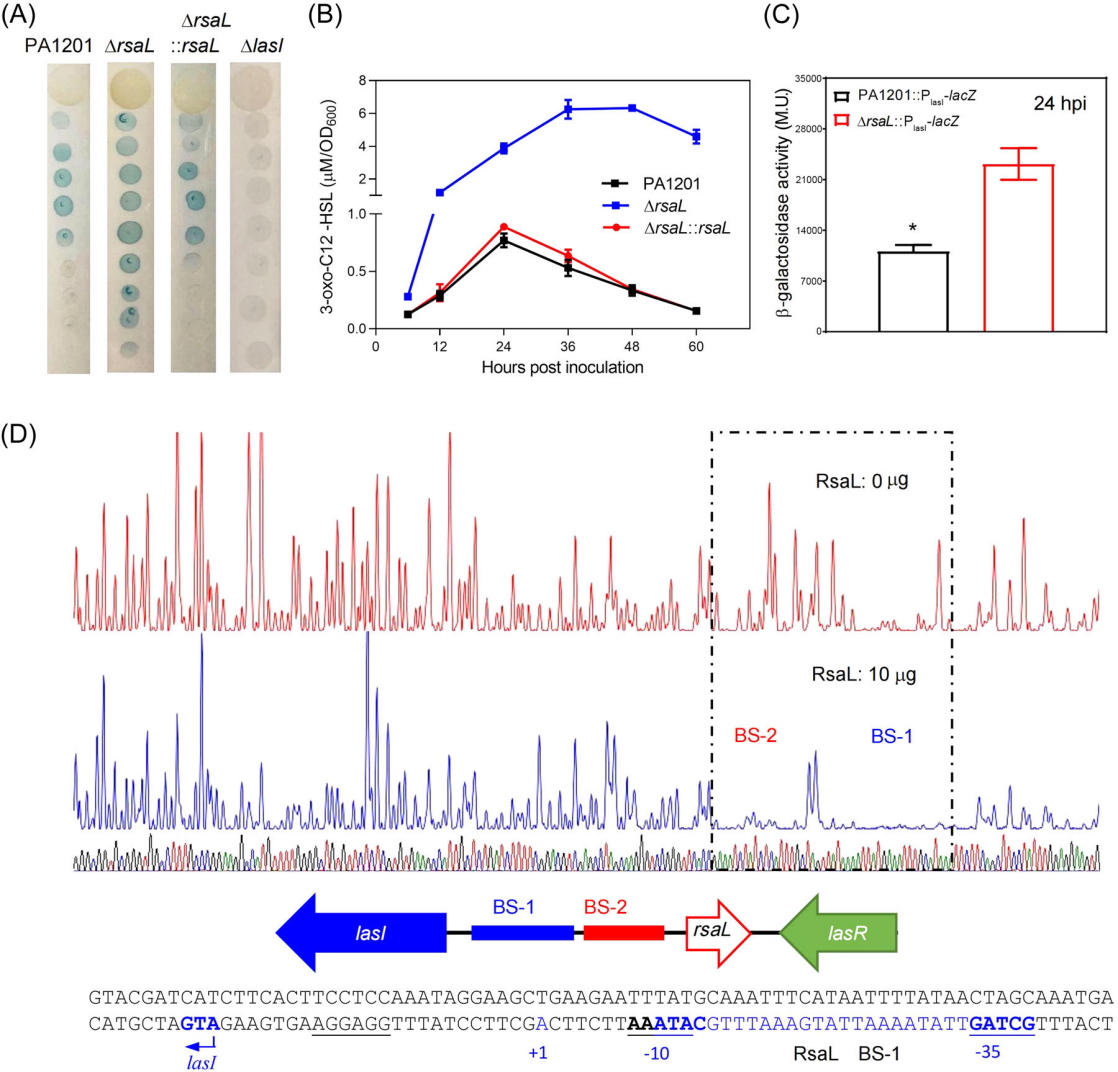
RsaL represses *lasI* transcription and 3‐oxo‐C12‐HSL biosynthesis. (A) Relative 3‐oxo‐C12‐HSL levels in the strains PA1201, Δ*rsaL*, Δ*rsaL*::*rsaL, and ΔlasL* evaluated with the biosensor strain CF11. (B) 3‐oxo‐C12‐HSL quantification in PA1201, Δ*rsaL*, and Δ*rsaL*::*rsaL* cultures by UPLC‐MS. (C) Relative transcriptional activity of the *LacZ* reporter gene under the control of the *lasI* promoter in the strains PA1201 and Δ*rsaL* at 24 hours postinoculation, measured as β‐galactosidase activity expressed in M.U. (D) Mapping of two RsaL‐binding sites (BS), with BS‐1 in the promoter P_lasI_ and BS‐2 in the promoter P_rsaL_ in the intergenic region between *lasI* and *rsaL*, by a DNase I footprint protection assay. The BS‐1 sequence corresponding to the RsaL binding sites in P_lasI_ is shown in blue. Three independent experiments were conducted; averages and standard deviations are shown. Statistically significant differences are indicated by one asterisk (*p* < 0.05).

To test the role of RsaL in the regulation of HSL‐dependent QS signals, elastase activity, and rhamnolipid production, we created the *rsaL* in‐frame deletion mutant Δ*rsaL*. In the PPM medium, this mutant produced more 3‐oxo‐C12‐HSL than wild‐type PA1201 during all growth phases (Figures 1C,D and 3A), but *rsaL* deletion had no significant effect on growth (Figure 1B). This result contrasted with the significant decrease in the 3‐oxo‐C12‐HSL level observed after 24 hpi in PA1201 (Figures 1C,D and 3B). 3‐oxo‐C12‐HSL overproduction in Δ*rsaL* could be complemented by integrating a single copy of *rsaL* in Δ*rsaL* (Figure 3A,B), confirming that RsaL acts as a repressor of 3‐oxo‐C12‐HSL production.

**Figure 4:**
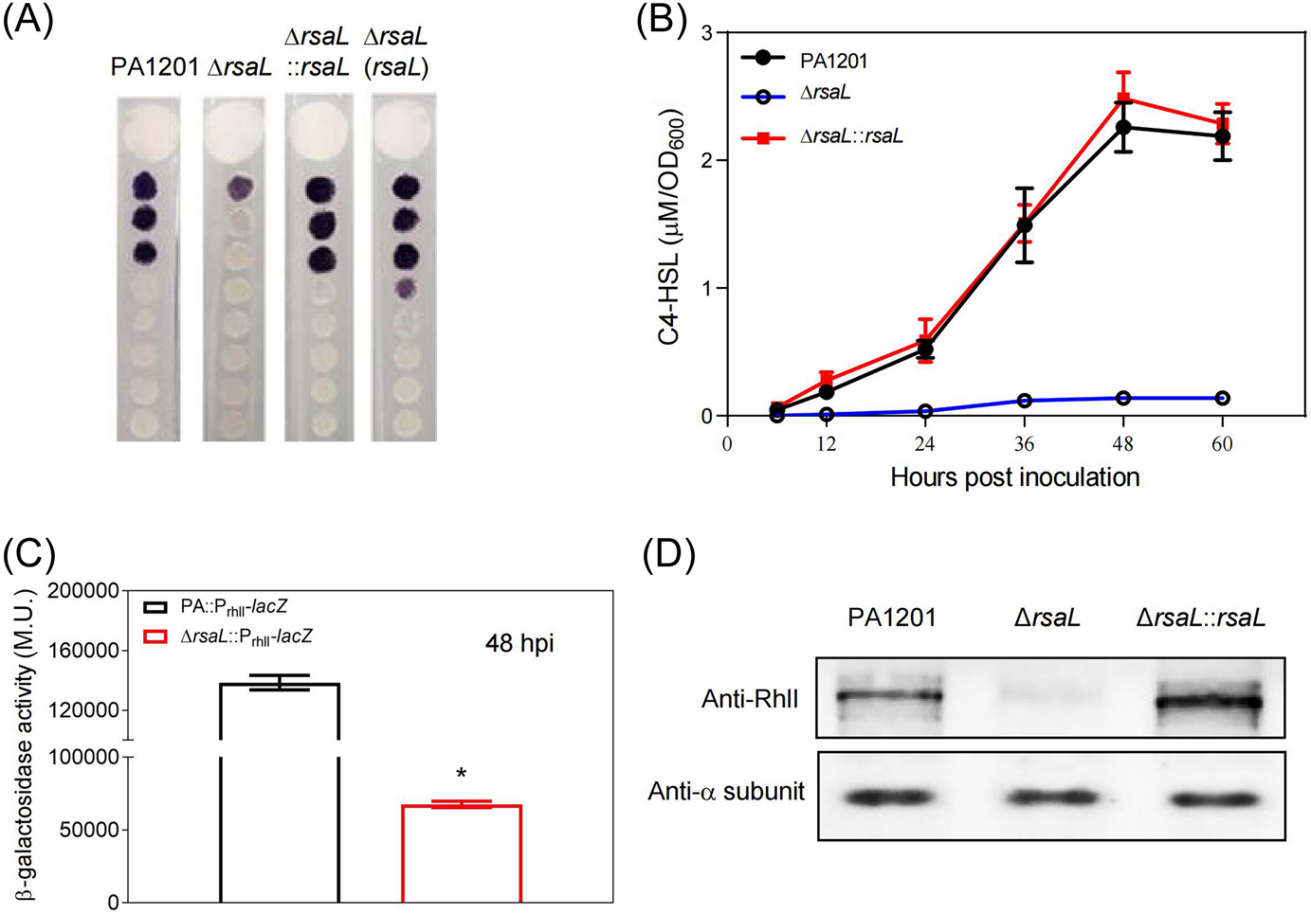
RsaL positively regulates C4‐HSL biosynthesis and *rhlI* expression. (A) Relative C4‐HSL levels in the strains PA1201, Δ*rsaL*, Δ*rsaL*::*rsaL*, and Δ*rsaL*(*rsaL)* evaluated with the biosensor strain CV026. (B) C4‐HSL quantification of PA1201, Δ*rsaL*, and Δ*rsaL*::*rsaL* cultures at different growth time points, measured by UPLC‐MS. (C) Relative transcriptional activity of the *LacZ* reporter gene under the control of the *rhlI* promoter in the strains PA1201 and Δ*rsaL* at 48 hours postinoculation, measured as β‐gal activity expressed in M.U. (D) RhlI protein levels in the strains PA1201, Δ*rsaL*, and Δ*rsaL*::*rsaL*. Three independent experiments were conducted; averages and standard deviations are shown. Statistically significant difference is indicated by one asterisk (*p* < 0.05).

When we evaluated C4‐HSL production in Δ*rsaL* cultured in PPM, we found that C4‐HSL production was almost completely abolished at all growth stages (Figures 1C,D and 4A,B). C4‐HSL production was restored by integrating a single copy of *rsaL* in Δ*rsaL*, demonstrating that RsaL is an essential activator of C4‐HSL production (Figure 4A,B). These results suggest that RsaL is a key regulator of the alternative biosynthesis of 3‐oxo‐C12‐HSL and C4‐HSL during PA1201 growth.

Next, we studied the effect of *rsaL* deletion on elastase activity and rhamnolipid production. *rsaL* deletion resulted in a dramatic increase in *lasB* expression level at the early growth stage (12–36 hpi) compared to the wild‐type expression level but had no significant effect at 48 hpi (Figure 2A). The increase in elastase activity caused by *rsaL* deletion at 24 hpi could be reversed by complementing Δ*rsaL* with a single copy of *rsaL* (Figure 2B).

At 12 and 24 hpi, *rhlA* expression in Δ*rsaL* and PA1201 was comparable. In contrast, at 36 and 48 hpi, Δ*rsaL* failed to upregulate *rhlA* expression, as in PA1201 (Figure 2C). Consistent with these results, at 48 hpi, *rhlA*‐dependent rhamnolipid production was significantly lower in Δ*rsaL* than in PA1201 but not at 24 hpi (Figure 2D). Complementation of Δ*rsaL* with a single copy of *rsaL* fully restored rhamnolipid production to the wild‐type level (Figure 2D). These results suggest that RsaL is also essential for controlling the alternative production of elastase and rhamnolipid during PA1201 growth.

### RsaL binds the –10 and –35 boxes in the *lasI* promoter (P_lasI_) to repress *lasI* transcription

RsaL has been shown to repress 3‐oxo‐C12‐HSL production by binding specifically an AT‐rich region (TATGAAATTTGCATA) within P_lasI_ in *P*. *aeruginosa* PAO1^8^. In this study, we generated the reporter strains PA1201::P_lasI_‐*lacZ* and Δ*rsaL*:: P_lasI_‐*lacZ* and tested the effect of *rsaL* deletion on *lasI* expression in PA1201. Indeed, at 24 hpi, P_lasI_‐dependent galactosidase activity significantly increased in Δ*rsaL* (Figure 3C), confirming that RsaL could repress *lasI* expression.

Using a DNA probe covering the *lasI*‐*rsaL* intergenic region, a DNase I footprint assay identified an RsaL‐binding site in P_lasI_ (BS‐1:ATACGTTTAAAGTATTAAAATATTGAT), which included a previously described RsaL‐binding site (ATACGTTTAAAGTAT) in PAO1, and covered the –10 and –35 boxes of P_lasI_ (Figure 3D). These results indicate that RsaL may repress *lasI* expression by binding BS‐1.

### RsaL activates *rhlI* transcription by binding a DNA region encoding the 5′‐untranslated region (UTR) of *rhlI* mRNA

Since *rsaL* was essential to C4‐HSL production (Figures 1D and 4A,B), we hypothesized that *rsaL* could control C4‐HSL production by activating *rhlI*. In keeping with this hypothesis, at 48 hpi, the *rhlI* promoter (P_rhlI_)‐dependent galactosidase activity in Δ*rsaL* (67,595 M.U.) was significantly lower than that in wild‐type PA1201 (138,547 M.U.) (Figure 4C). This result was confirmed at the protein level by western blot, using in‐house generated polyclonal antibodies against RhlI to compare RhlI expression in wild‐type PA1201, Δ*rsaL*, and Δ*rsaL*::*rsaL* (Figure 4D).

**Figure 5:**
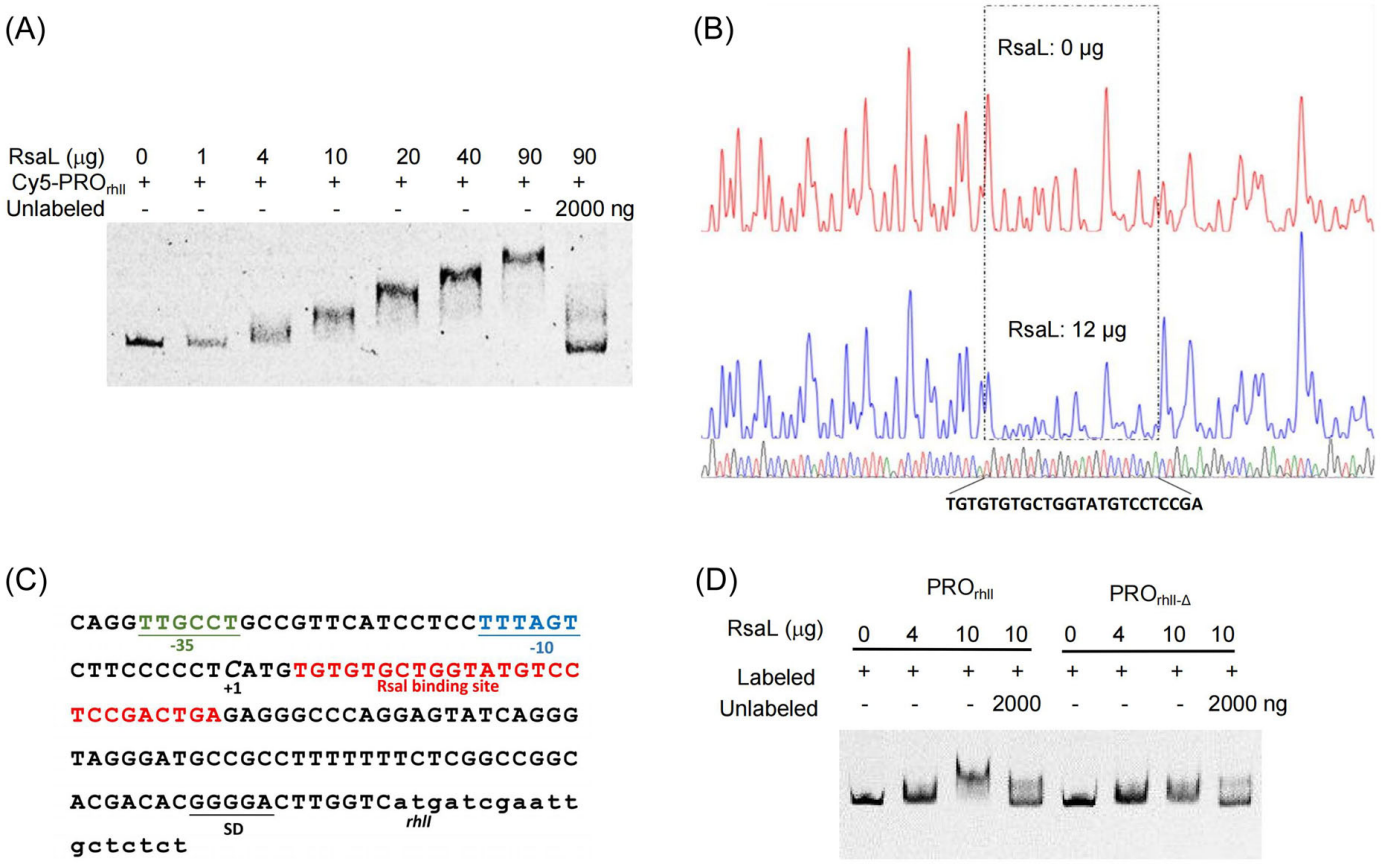
RsaL directly binds a DNA region encoding the 5′ untranslated region of *rhlI* mRNA. (A) Electrophoretic mobility shift assay (EMSA) results showing that RsaL binds the probe PRO_rhlI_, encompassing the *rhlI* promoter. 2000 ng of unlabeled probe was added as a specific competitor. (B) The RsaL binding site in PRO_rhlI_ identified by a DNase I protection assay. (C) DNA sequence of the *rhlI* promoter region; the RsaL‐binding site is shown in red and is located in the region encoding the 5′ untranslated region of *rhlI* mRNA. (D) EMSA results comparing RsaL‐binding capacity to wild‐type PRO_rhlI_ and PRO_rhlI‐Δ_, carrying a deletion of the RsaL putative binding site in the *rhlI* promoter. EMSA, electrophoretic mobility shift assay.

**Figure 6:**
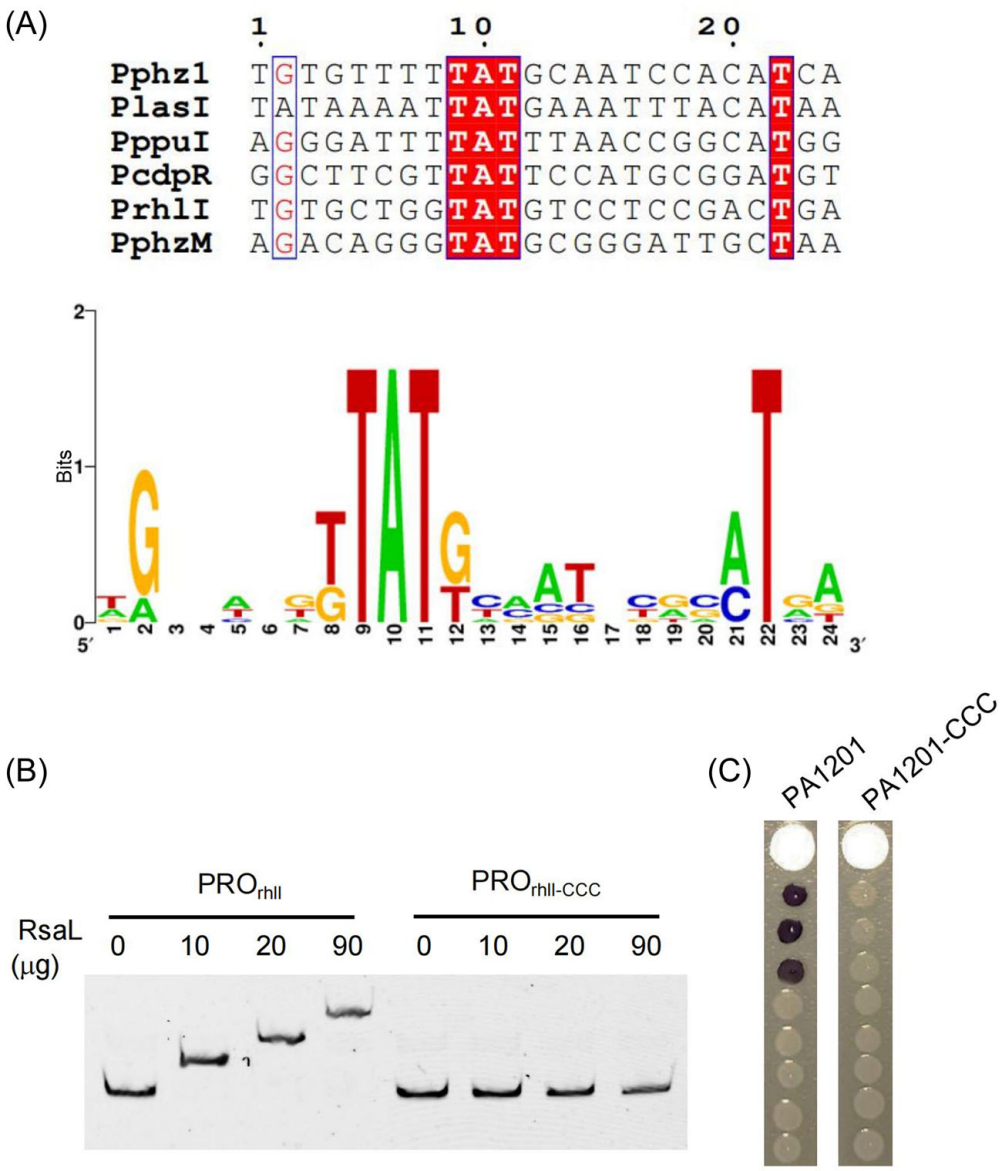
Functional characterization of the RsaL binding site in the DNA region encoding the 5′‐UTR of *rhlI* mRNA. (A) Alignment analysis of the *rhlI* promoter (P_rhlI_) with other gene promoters containing an RsaL‐binding motif and identification of the conserved residues defining a consensus RsaL binding site. (B) Impairment of RsaL binding to the probe PRO_rhlI_ mutated in the RsaL putative binding site (PRO_rhlI‐ccc_), demonstrated by EMSA. (C) Relative C4‐HSL levels in wild‐type PA1201 and PA1201 carrying the CCC mutation in P_rhlI_ (PA1201‐CCC) evaluated by the biosensor strain CV026.

Electrophoretic mobility shift assay (EMSA) showed that RsaL could bind a 227‐bp DNA probe, PRO_rhlI_, covering the *rhlI* promoter P_rhlI_ (Figure 5A). Further DNase I footprint analysis identified a 26‐bp RsaL‐binding site, TGTGTGCTGGTATGTCCTCCGACTGA, within the probe PRO_rhlI_ (Figure 5B,C). When this 26‐bp DNA sequence was deleted from PRO_rhlI_ to generate the variant probe PRO_rhlI‐Δ_, RsaL lost the ability to bind PRO_rhlI‐Δ_ (Figure 5D). Previously, RsaL has been shown to bind the promoter region of several genes, including *lasI*, *phzM*, and *phz1* ^8^, ^14^, ^18^. Multiple sequence alignment analyses of known RsaL binding sites identified an AT‐rich core sequence (Figure 6A). Point mutations of the conserved TAT into CCC within the PRO_rhlI_ resulted in a loss of RsaL‐binding activity (Figure 6B). Consistently, point mutations of the conserved TAT into CCC in the PA1201 genome led to a loss in C4‐HSL production in the mutated strain PA1201‐CCC (Figure 6C).

As shown in Figure 5C, P_rhlI_ has been well characterized^26^. The RsaL binding site identified in the present study is located within the DNA region encoding the 5′‐UTR of *rhlI* mRNA (Figure 5C), raising the question of whether RsaL could be an mRNA‐binding protein. To address this question, we generated Cy5‐labeled single‐stranded DNA or mRNA fragments corresponding to the probe PRO_rhlI_. EMSA analysis revealed that RsaL had no binding activity to single‐stranded DNAs or mRNAs derived from PRO_rhlI_ (Figure S1). Taken together, these results demonstrate that RsaL binds the double‐stranded DNA sequence corresponding to the 5′‐UTR of *rhlI* mRNA to positively regulate *rhlI* transcription.

### RsaL regulation of C4‐HSL biosynthesis is independent of the Las and PQS QS systems in PA1201

**Figure 7:**
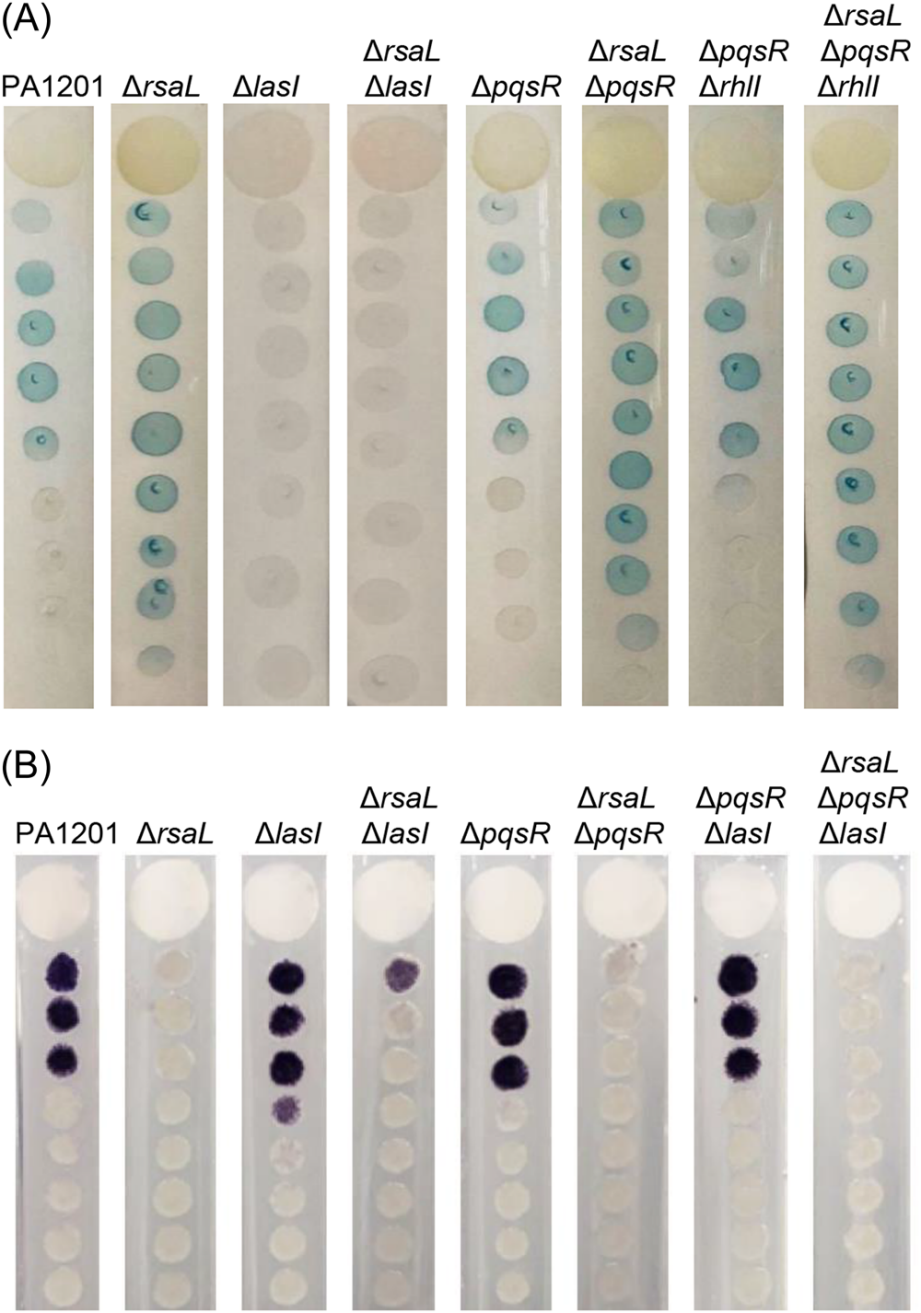
The RsaL‐dependent regulation of C4‐HSL biosynthesis is independent of other Las and PQS quorum sensing systems. C4‐HSL (A) and 3‐oxo‐C12‐HSL (B) level in PA1201, Δ*rsaL*, Δ*lasI*, Δ*lasI*Δ*rsaL*, Δ*pqsR*, Δ*pqsR*Δ*rsaL*, Δ*lasI*Δ*pqsR*, Δ*rhlI*Δ*pqsR*, Δ*rhlI*Δ*pqsR*Δ*rsaL*, and Δ*lasI*Δ*pqsR*Δ*rsaL*, were evaluated with the biosensor strain CV026 (A) and CF11 (B), respectively.

PA1201 possesses at least three QS signaling pathways that regulate its virulence and adaptation to different environmental conditions^27^. To determine whether the RsaL‐dependent regulation of C4‐HSL biosynthesis is regulated by the Las and PQS QS systems, we generated a range of QS gene‐deleted mutants. As expected, 3‐oxo‐C12‐HSL depended on LasI activity, as the Δ*lasI* and the Δ*lasI*Δ*rsaL* strains failed to produce 3‐oxo‐C12‐HSL (Figure 7A). In contrast, deleting *pqsR* had no effect on 3‐oxo‐C12‐HSL biosynthesis. Deletion of *rsaL* in the Δ*pqsR* or Δ*pqsR*Δ*rhlI* strains significantly increased 3‐oxo‐C12‐HSL biosynthesis (Figure 7A). These results suggest that RsaL‐dependent repression of 3‐oxo‐C12‐HSL biosynthesis is independent of the Rhl and PQS pathways.

Δ*lasI* produced a similar level of C4‐HSL to that produced by wild‐type PA1201, while Δ*rsaL*Δ*lasI* produced a similar level of C4‐HSL to that produced by Δ*rsaL* (Figure 7B). In the same way, deleting *pqsR*, a key regulator of the PQS pathway, had no effect on C4‐HSL biosynthesis. Deleting *rsaL* in the strains Δ*pqsR* or Δ*pqsR*Δ*lasI* resulted in disrupted C4‐HSL biosynthesis (Figure 7B). These results suggest that the RsaL‐dependent regulation of C4‐HSL biosynthesis is independent of the Las and PQS signaling systems.

### RsaL acts as an auto‐repressor in PA1201

**Figure 8:**
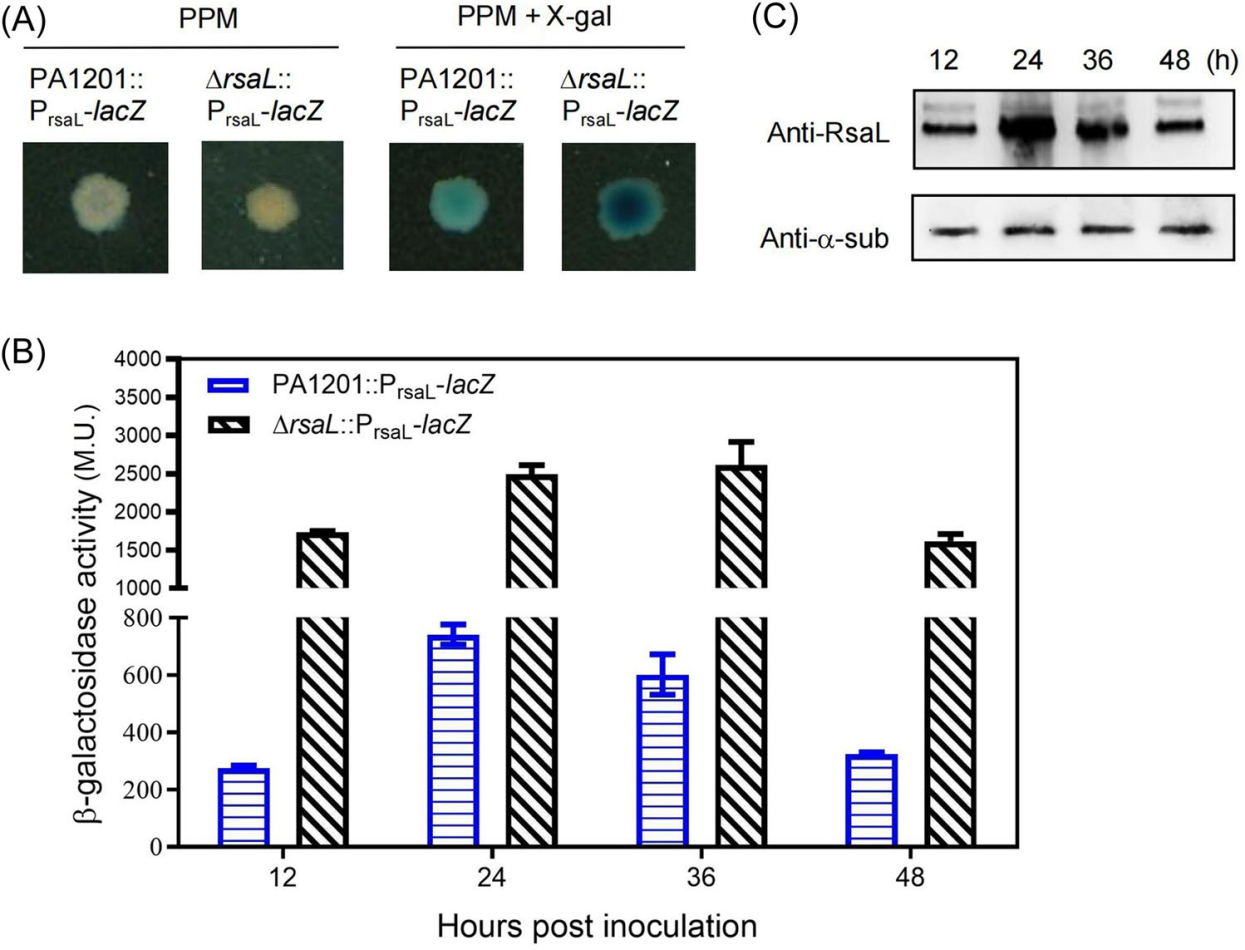
Kinetics of *rsaL* expression during PA1201 growth. (A) Verification of the reporter strains PA1201::P_rsaL_ *‐lacZ* and Δ*rsaL*::P_rsaL_ *‐lacZ* on the PPM agar plate supplemented or not with the β‐galactosidase substrate X‐gal. (B) Kinetics of *rsaL* expression during PA1201 growth, estimated by measuring β‐galactosidase activity expressed in M.U. Data are expressed as means ± standard deviations obtained in three independent assays. (C) Kinetics of RsaL protein production in PA1201 grown in PPM medium assessed by western blot using in‐house polyclonal antibodies against RsaL (upper blot) and a commercially available antibody against the α‐subunit of RNA polymerase as a control for sample loading (lower blot).

To monitor *rsaL* expression during growth, the *rsaL* promoter (P_rsaL_) was cloned upstream of the *lacZ* coding sequence to generate the reporter plasmid P_rsaL_‐*lacZ* subsequently integrated into wild‐type PA1201 and Δ*rsaL* to generate the reporter strains PA1201::P_rsaL_‐*lacZ* and Δ*rsaL*::P_rsaL_‐*lacZ* (Figure 8A). PA1201::P_rsaL_‐*lacZ* displayed P_rsaL_‐dependent galactosidase activity that was significantly higher at 24 hpi (741.5 M.U.) and 36 hpi (602 M.U.) than at 12 hpi (275.5 M.U.) and 48 hpi (324 M.U.) (Figure 8B). To confirm the correlation between the gene reporter activity and the presence of the RsaL protein, we generated polyclonal antibodies (anti‐RsaL) against a recombinant RsaL protein produced in *Escherichia* *coli* and purified by chromatography (Figure S2). Using this new antibody, we confirmed that the RsaL protein level at 24 and 36 hpi was significantly higher than that at 12 and 48 hpi when PA1201 was cultured in PPM (Figure 8C).

Interestingly, the Δ*rsaL*::P_rsaL_‐*lacZ* reporter strain, deprived of RsaL, displayed significantly higher P_rsaL_‐dependent galactosidase activity than PA1201::P_rsaL_‐*lacZ* (Figure 8A,B). Thus, RsaL negatively regulates its own expression in PA1201.

### RsaL binds to the −35 box of the *rsaL* gene promoter P_rsaL_

**Figure 9:**
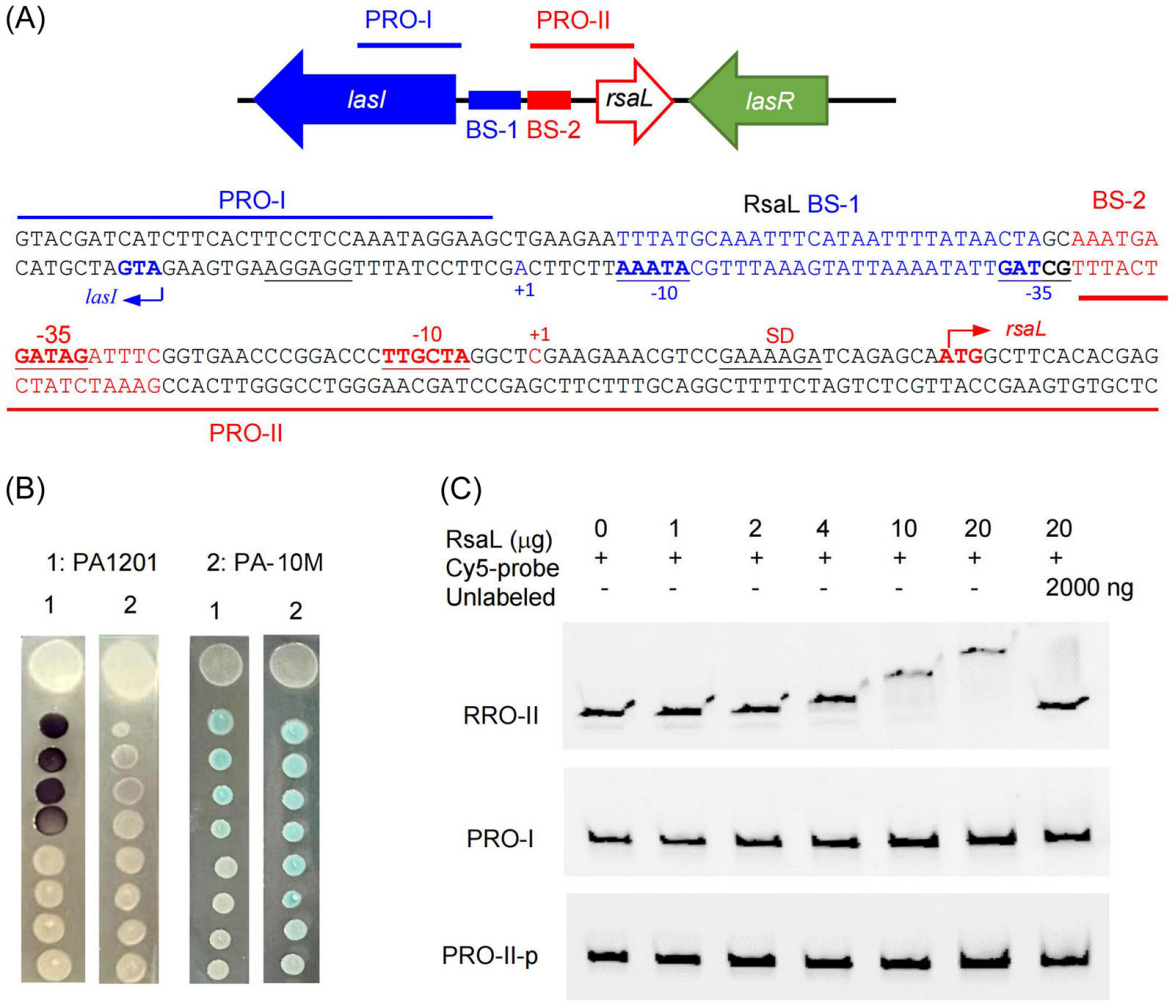
RsaL binding in the *rsaL* promoter (P_rsaL_). (A) Analysis of the promoters located in the intergenic region between *lasI* and *rsaL* in PA1201. The *lasR*, *rsaL*, and *lasI* genes are depicted as arrows. The corresponding sequence is annotated for the RsaL binding sites and the –10 and –35 boxes of the *rsaL* and *lasI* promoters. (B) Assessment of 3‐oxo‐C12‐HSL (left) and C4‐HSL (right) production using the biosensors CF11 (left) and CV026 (right) in wild‐type PA1201 and PA‐10M mutated in the –10 box of the *rsaL* promoter (P_rsaL_). (C) EMSA results showing the functionality of the RsaL binding site in BS‐2. The probes PRO‐I and PRO‐II are shown in (A). PRO‐II‐p corresponds to PRO‐II, with a point mutation in the RsaL binding site 2 located in the –35 box of P_rsaL_.

To study the molecular mechanism of RsaL self‐repression, first, we characterized P_rsaL_ by rapid amplification of cDNA ends (RACE) analysis. This experiment identified the transcription start site (+1, C) in P_rsaL_ (Figure 9A). Further promoter prediction using the SAPPHIRE program identified the –10 box (TTGCTA) and the –35 box (GATAGA) in P_rsaL_ (Figure 9A). The functionality of this predicted promoter was tested by mutagenesis. The –10 box TTGCTA was mutated into CCGCTA on the PA1201 chromosome. The resulting mutant, PA‐10M, displayed a phenotype similar to Δ*rsaL* (i.e., increased 3‐oxo‐C12‐HSL biosynthesis and disrupted C4‐HSL production; Figure 9B). These results confirmed that the predicted –10 box was constitutive of P_rsaL_ (Figure 9A).

Next, we investigated the potential binding sites of RsaL on P_rsaL_ using a DNase I protection footprint assay on both strands of a DNA fragment encompassing the entire *rsaL*‐*lasI* intergenic region. In addition to BS1 (Figure 3D), this analysis identified a second binding site, BS‐2 (AAATGAGATAGATTTC), which could correspond to the RsaL binding site responsible for P_rsaL_ regulation. BS‐2 partially overlapped the –35 box of P_rsaL_ and contained a palindromic sequence (Figure 9C). This symmetrical motif suggests that RsaL could bind P_rsaL_ as a dimer. The functionality of this binding site was assessed in EMSA experiments using the Cy5‐labeled probe I (PRO‐I), excluding BS‐1 and BS‐2, and the Cy5‐labeled probe II (PRO‐II), encompassing BS‐2 and flanking sequences but excluding BS‐1 (Figure 9A). RsaL bound only PRO‐II and not PRO‐I (Figure 9C). Mutation of the BS‐2 palindromic sequence ATTTC into GCCCC (PRO‐II‐p) disrupted RsaL binding to PRO‐II (Figure 9C), demonstrating that this DNA motif was responsible for BS‐2 recognition by RsaL.

## DISCUSSION

The *P. aeruginosa* strain PA1201 has become a model rhizosphere bacterium for studying the regulation of PCA or PCN biosynthesis^12^, ^13^, ^14^. Our previous research showed that PA1201 possesses all three QS pathways and differentially regulates PCA biosynthesis^27^. Here, we further show that when PA1201 is cultured in PPM, the 3‐oxo‐C12‐HSL level plateaus at 24 hpi and then drops. In contrast, the C4‐HSL level is low at 12 hpi and starts to increase from 24 hpi onward, reaching peak production at 48 hpi (Figure 1). These alternative productions are correlated with the highest elastase activity at 24 hpi and the highest rhamnolipid level at 48 hpi (Figure 2). These results are consistent with previous findings in PAO1, in which the 3‐oxo‐C12‐HSL level plateaued in the late logarithmic growth phase, whereas the C4‐HSL level continued to increase^8^. Thus, *P. aeruginosa* has the ability to coordinate the alternative biosynthesis of 3‐oxo‐C12‐HSL and C4‐HSL during growth. To explore the underlying mechanism of this regulation, Sun et al.^14^ proposed a hypothesis based on substrate competition. Since the lactone rings of 3‐oxo‐C12‐HSL and C4‐HSL both derive from the same precursor, *S*‐adenosylmethionine, excessive production of 3‐oxo‐C12‐HSL at the early growth stage likely drains the pool of *S*‐adenosylmethionine necessary for C4‐HSL biosynthesis, thus leading to reduced C4‐HSL production^14^. However, the present study demonstrated for the first time that the transition from 3‐oxo‐C12‐HSL to C4‐HSL QS signals is regulated by an intrinsic mechanism involving the global regulator RsaL. The RsaL‐dependent regulation of 3‐oxo‐C12‐HSL and C4‐HSL biosynthesis is independent of the Las and PQS QS systems (Figure 7). Nevertheless, we could not rule out the minor role of substrate competition in the regulation of 3‐oxo‐C12‐HSL and C4‐HSL biosynthesis in PA1201.

Previous results have shown that RsaL is required for 3‐oxo‐C12‐HSL homeostasis^8^. The present study further suggests that RsaL is also essential for C4‐HSL homeostasis. Deletion of *rsaL* abolished C4‐HSL biosynthesis in PA1201 (Figures 1 and 4). RsaL activated *rhlI* expression by binding a DNA region encoding the 5′‐UTR of *rhlI* mRNA (Figure 5). However, we could not find evidence that RsaL could directly interact with single‐stranded DNAs or mRNAs corresponding to *rhlI* 5′‐UTR. How RsaL could have antagonistic effects on DNA binding and its biological significance deserves further investigation.

Thomason et al.^26^ conducted a term‐seq analysis of PAO1 and identified an sRNA named RhlS, which is derived from the 5′‐UTR of *rhlI*. RhlS is required for the production of normal C4‐HSL levels by promoting *rhlI* translation. An antisense RNA (asRhlS) to the 5′‐terminal of the *rhlI* open reading frame has also been identified in PAO1. RhlS may act antagonistically to the asRhlS to regulate *rhlI* translation^26^. The experiments in the present study could not detect binding activity between RsaL and single‐stranded *rhlI* fragments or *rhlI* mRNA (Figure S1). Whether RsaL regulates the transcription of RhlS or asRhlS and whether two small RNAs mediate the RsaL regulation of 3‐oxo‐C12‐HSL and C4‐HSL biosynthesis deserve further study.

**Figure 10:**
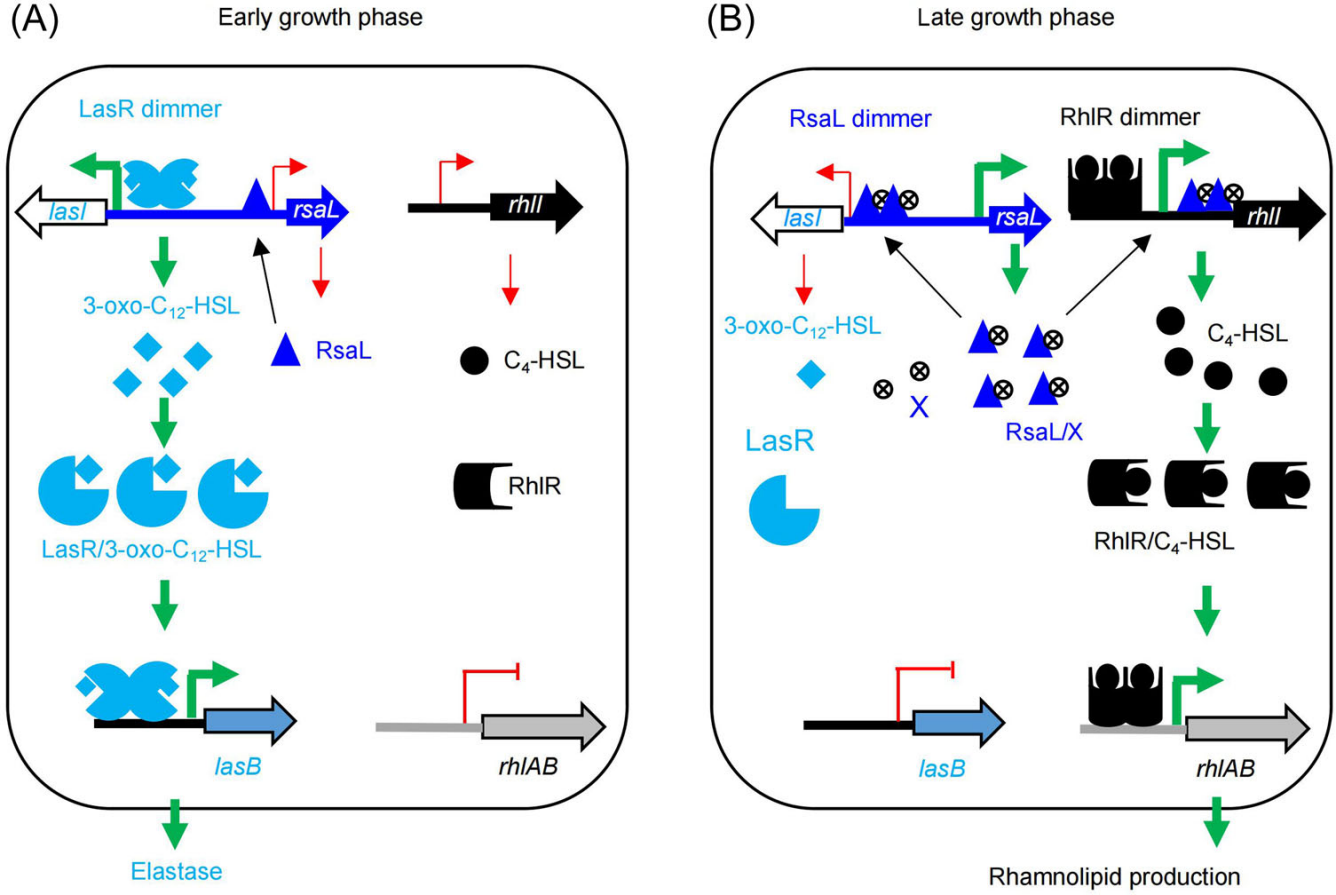
Model for the RsaL‐dependent control of differential AHL QS‐dependent virulence in *Pseudomonas aeruginosa* PA1201. During the early growth phase (A), *rsaL* is transcribed at a low level; RsaL binds the –35 box and represses its own transcription; no RsaL binds the P_lasI_ promoter, and *lasI* is activated by the 3‐oxo‐C12‐HSL‐dependent LasR system, while *lasB* transcription and elastase activity are activated by the Las QS system. *rhlI* is transcribed at a low level, which is not enough to promote C4‐HSL‐dependent rhamnolipid biosynthesis. During the late growth phase (B), a signal molecule X, as yet to be identified, informs the bacteria that a population threshold has been reached, could bind RsaL and release it from P_rsaL_, thereby increasing *rsaL* transcription. In parallel, RsaL/X complexes could outcompete LasR/3‐oxo‐C12‐HSL complex on P_lasI_ and repress *lasI* transcription. RsaL/X complex could also bind to the DNA region encoding the 5′‐UTR of the *rhlI* promoter, inducing *rhlI* transcription, which in turn induces the transcription of the *rhlAB* cluster for rhamnolipid production. QS, quorum sensing.

Taking these results together, we propose a new hypothetical model of QS regulation, integrating RsaL autorepression and RsaL‐dependent regulation of 3‐oxo‐C12‐HSL and C4‐HSL biosynthesis in PA1201. During the early growth phase (Figure 10A), *rsaL* is transcribed at a basal low level, RsaL binds the –35 box of its own promoter, which limits *rsaL* transcription, no RsaL binds the P_lasI_ promoter, and *lasI* transcription is induced via a 3‐oxo‐C12‐HSL‐dependent LasR system. *lasB* transcription is induced by the Las QS system, which promotes high elastase activity. *rhlI* is transcribed at a basal level that is not sufficient to induce C4‐HSL‐dependent rhamnolipid biosynthesis. During the late growth phase (Figure 10B), a signal molecule X, as yet to be identified, which informs the bacteria that a population threshold has been reached, could serve as a ligand for RsaL. The RsaL/X complex could release RsaL from P_rsaL_, which would increase *rsaL* transcription and result in a high level of RsaL. In parallel, RsaL/X complexes could outcompete the LasR/3‐oxo‐C12‐HSL complex for binding to P_lasI_ and repress *lasI* transcription. The RsaL/X complex could also bind the DNA region encoding the 5′‐UTR of the *rhlI* promoter, inducing *rhlI* transcription, which in turn could induce the transcription of the *rhlAB* cluster for rhamnolipid production.

## MATERIALS AND METHODS

### Bacterial strains and growth conditions

All the bacterial strains used in this study are listed in Table S1. Unless stated otherwise, PA1201 and all isogenic mutants were grown at 28°C in 50 ml of PPM (22 g/l tryptone, 20 g/l glucose, 5 g/l KNO_3_, pH 7.5) in 250‐ml flasks. *E. coli* strains were grown in shake culture at 37°C in Luria–Bertani broth. When required, the following antibiotics were added to the media: spectinomycin (Spe, 50 µg/ml), kanamycin (Kan, 50 µg/ml), and gentamycin (Gen, 100 µg/ml for *P. aeruginosa* strains and 20 µg/ml for *E. coli* strains).

### DNA engineering and generation of in‐frame deletion mutants

DNA preparation and agarose gel electrophoresis were performed following the protocols described by Sambrook and Russell^28^. The plasmids and oligonucleotides used in this study are listed in Tables S2 and S3. The gene deletion mutants of PA1201 were generated as previously described^29^. Briefly, the ∼500 bp upstream and downstream regions of the target gene were fused by PCR. The PCR product was then subcloned into the plasmid pK18mobsacB. The resultant recombinant plasmid was further integrated within the target gene of XC1. The resultant strain was finally plated on an NYG agar plate with 50 μg/ml Spe and 5% (w/v) sucrose. The generated mutants were verified by colony PCR and subsequent DNA sequencing. Single‐copy gene complementation was performed following the protocol described by Jittawuttipoka et al.^30^ Briefly, the fragment corresponding to the promoter and coding region of a target gene was amplified and cloned into a mini‐Tn7T‐Gm transposon, which was subsequently integrated into PA1201 at the neutral site attTn7.

### Construction of the promoter‐*lacZ* fusion reporter strain and β‐galactosidase assays

Construction of *lacZ*‐dependent reporter strains in PA1201 was conducted following a previously described protocol^25^. Briefly, approximately 500 bp promoter regions with 30 bp coding sequences from the target genes were PCR‐amplified and cloned into mini‐CTX‐lacZ vectors^31^. The resulting plasmids were integrated into PA1201‐derived strains. β‐Galactosidase activity of the constructed reporter strains was determined according to the protocol described by Miller^28^.

### Protein expression and purification

RsaL protein expression and purification has been previously described^14^. Briefly, the *rsaL* gene was amplified by PCR and then cloned into the plasmid pET28a. The resulting plasmid was introduced into *E. coli* BL21 (DE3, pLysS). RsaL expression was induced with 0.1 mM isopropyl β‐d‐1‐thiogalactopyranoside at OD_600_ = 0.6, and the culture was grown in a 16°C shaker for 12 h. The recombinant protein was purified by Ni^2+^‐affinity chromatography according to the manufacturer's instructions. Briefly, bacterial cells were lysed in the lysis buffer with 10 mM Tris‐buffer saline, 100 mM NaCl, 100 mM (NH_4_)_2_SO_4_, and 10 mM imidazole, pH 7.2. His‐tagged RsaL proteins were eluted in an elution buffer with 10 mM Tris‐buffer saline, 100 mM NaCl, 100 mM (NH_4_)_2_SO_4_, and 250 mM imidazole, pH 7.2. The eluted RsaL protein was finally loaded into a Superdex 200 gel filtration column (GE Healthcare) and equilibrated with a nonimidazole buffer containing 10 mM Tris‐HCl, 150 mM NaCl, 10% (v/v) glycerol, pH 7.5.

### EMSA

EMSA was performed using the Thermo Scientific Light Shift Chemiluminescent EMSA Kit. Briefly, the probe DNA fragments were biotin‐labeled at the 5′‐end. The labeled probes were then incubated with RsaL protein in binding buffer (10 mM Tris‐HCl, 1 mM ethylenediaminetetraacetic acid, 5 mM MgCl_2_, 50 mM KCl, 2.5% [v/v] glycerol, 30 μg/ml poly(dI‐dC), 0.05% [v/v] NP‐40, pH 8.0). After incubation at room temperature for 20 min, the reaction mixtures were loaded on a 5% (w/v) polyacrylamide gel under nondenaturing conditions. The PAGE gel was then submitted for scanning for fluorescent DNA using a Starion FLA‐9000 Scanner (FujiFilm).

### DNase I protection footprint sequencing

DNase I protection footprint sequencing was performed at Shanghai Biotechnology Corporation. Briefly, the fluorescent probes were PCR amplified. The products were then purified using a PCR Clean‐Up System (Promega). 100 ng of probe was incubated with different amounts of RsaL in a total volume of 40 µl reaction mixtures. After incubation for 30 min at room temperature, 10 µl of the solution containing 0.015 units of DNase I (Promega) and 100 nM CaCl_2_ was added, and the reaction mixtures were further incubated for 1 min at 25°C. The reaction was terminated by adding 140 µl of stop solution. Samples were extracted with phenol/chloroform, precipitated with ethanol, and the pellets were dissolved in 30 µl of water. Gel electrophoreses were performed and the data were analyzed using a 3130XL DNA analyzer and the Peak Scanner Software v1.0 (Applied Biosystems).

### Quantitative determination of 3‐oxo‐C12‐HSL and C4‐HSL levels using biosensor strains

The synthesis of C4‐HSL and 3‐oxo‐C12‐HSL in PA1201 and derived mutants was assessed by semiquantitative diffusion plate assay using *C. violaceum* strain CV026 and *A. tumefaciens* CF11 biosensors, respectively, as previously described^23^. Purple or blue spots indicated that the diffusible QS signal molecules were detected by the biosensor strains. The relative level of C4‐HSL or 3‐oxo‐C12‐HSL was proportional to the diffusion distance of the QS molecules in PPM cultures of their respective sensor strains.

### Extraction and quantification of 3‐oxo‐C12‐HSL and C4‐HSL by UPLC‐MS

3‐oxo‐C12‐HSL and C4‐HSL extraction and quantification were performed following a previously described protocol^14^. Briefly, 270 µl of culture with an OD_600_ of 1.8–6.9 was acidified to pH 4.0 with 6 M HCl, and extracted with an equal volume of ethyl acetate. A total of 100 µl of ethyl acetate extract was evaporated at 40°C, and the resulting residue was dissolved in 500 µl of methanol. Then 10 μl of the extract was then injected into an UPLC‐MS (Agilent UPLC1290‐TOF‐MS6230), under the following conditions: Agilent Zorbax XDB C18 reverse‐phase (5 µm, 4.6 × 150 mm) system separated by gradient ACN with 0.5% acetic acid and H_2_O with 0.5% (v/v) acetic acid at 0.4 ml/min. The MS analysis was performed under positive mode. The concentration of 3‐oxo‐C12‐HSL and C4‐HSL was quantified using the peak area (A) of the specific extracted ion chromatogram in the total ion chromatogram according to the established formula^14^.

### Western blot analysis

RsaL proteins were electrotransferred onto a polyvinylidene difluoride membrane (Roche). After blocked with 5% (w/v) nonfat milk powder, the membranes were incubated with the rabbit polyclonal antibodies against RsaL at a 1:3000 dilution. The membrane was then washed three times with TBST buffer (20 mM Tris, 0.15 M NaCl, and 0.1% [v/v] Tween 20). A horseradish peroxidase‐conjugated goat antirabbit IgG (#M21001; Abmart) diluted at 1:6500 was used as the secondary antibody. After membrane washing, the luminescent signal was detected using an ECL kit and a ChampChemi 610 Plus instrument (Sage Creation Science).

### Mapping of *rsaL* transcription start site

The transcription start site of *rsaL* was identified using the 5′‐RACE system (Invitrogen). Briefly, total RNA was prepared from PA1201 PPM culture at 24 hpi. The cDNA synthesis was performed following the manufacturer's protocol. After cDNA synthesis, RNase was added to remove the residual RNA. An oligo‐dC tail was added to the generated cDNA. The dC‐tailed cDNA was amplified by PCR and was then cloned into the pGEM‐T Easy vector for sequencing.

### Statistical analysis

All of the experiments were performed in triplicate. The statistical significance of the differences observed in mean invasion frequency was determined by calculating the *p* values using the two‐tailed Student *t‐*test for unpaired data sets.

## AUTHOR CONTRIBUTIONS

**Ya‐Wen He**: Conceptualization (equal); data curation (equal); formal analysis (equal); funding acquisition (equal); investigation (equal); methodology (equal); project administration (equal); supervision (equal); validation (equal); writing—original draft (equal); writing—review and editing (equal). **Zi‐Jing Jin**: Investigation (equal). **Ying Cui**: Investigation (equal). **Kai Song**: Data curation (supporting); formal analysis (supporting). **Bo Chen**: Investigation (supporting). **Lian Zhou**: Conceptualization (supporting); investigation (supporting).

## ETHICS STATEMENT

There is no animal or human used in this study.

## CONFLICT OF INTERESTS

The authors declare no conflict of interests.

## Supporting information

Supporting information.

## Data Availability

The data that support the findings of this study are available from the corresponding authors upon reasonable request.
