# Supplementary material for: RsaL is a self‐regulatory switch that controls alternative biosynthesis of two AHL‐type quorum sensing signals in Pseudomonas aeruginosa PA1201
Source: mLife. 2024 Mar 18;3(1):74–86. doi: 10.1002/mlf2.12113 (PMC11139201; doi:10.1002/mlf2.12113)
Supplement: Supplementary file 1 — Supporting information. [file MLF2-3-74-s001.ppt]

## Slide 1
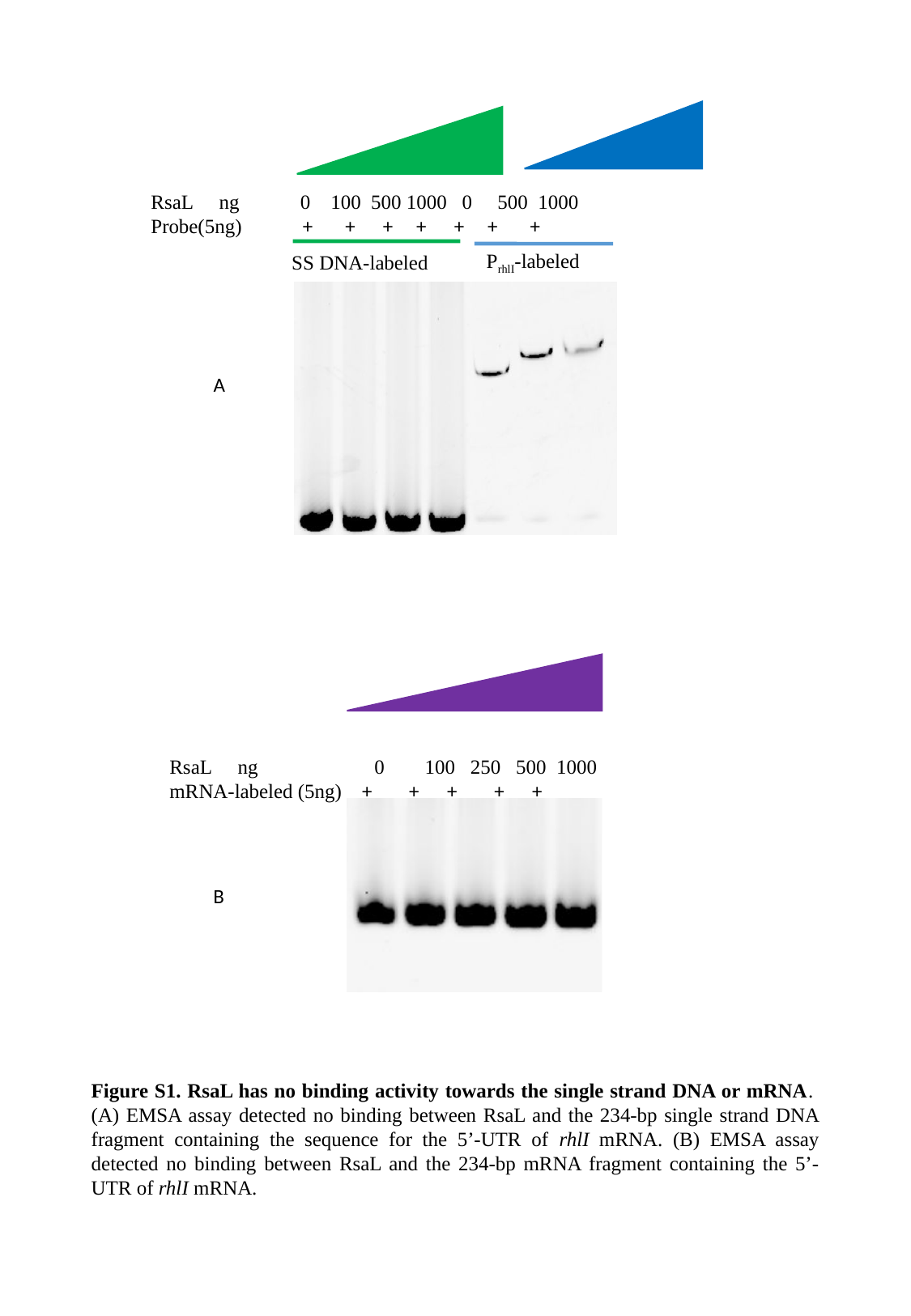

RsaL（ng） 0 100 500 1000 0 500 1000
Probe(5ng) + + + + + + +
PrhlI-labeled
SS DNA-labeled
A
RsaL（ng） 0 100 250 500 1000
mRNA-labeled (5ng) + + + + +
B
Figure S1. RsaL has no binding activity towards the single strand DNA or mRNA. (A) EMSA assay detected no binding between RsaL and the 234-bp single strand DNA fragment containing the sequence for the 5’-UTR of rhlI mRNA. (B) EMSA assay detected no binding between RsaL and the 234-bp mRNA fragment containing the 5’-UTR of rhlI mRNA.

## Slide 2
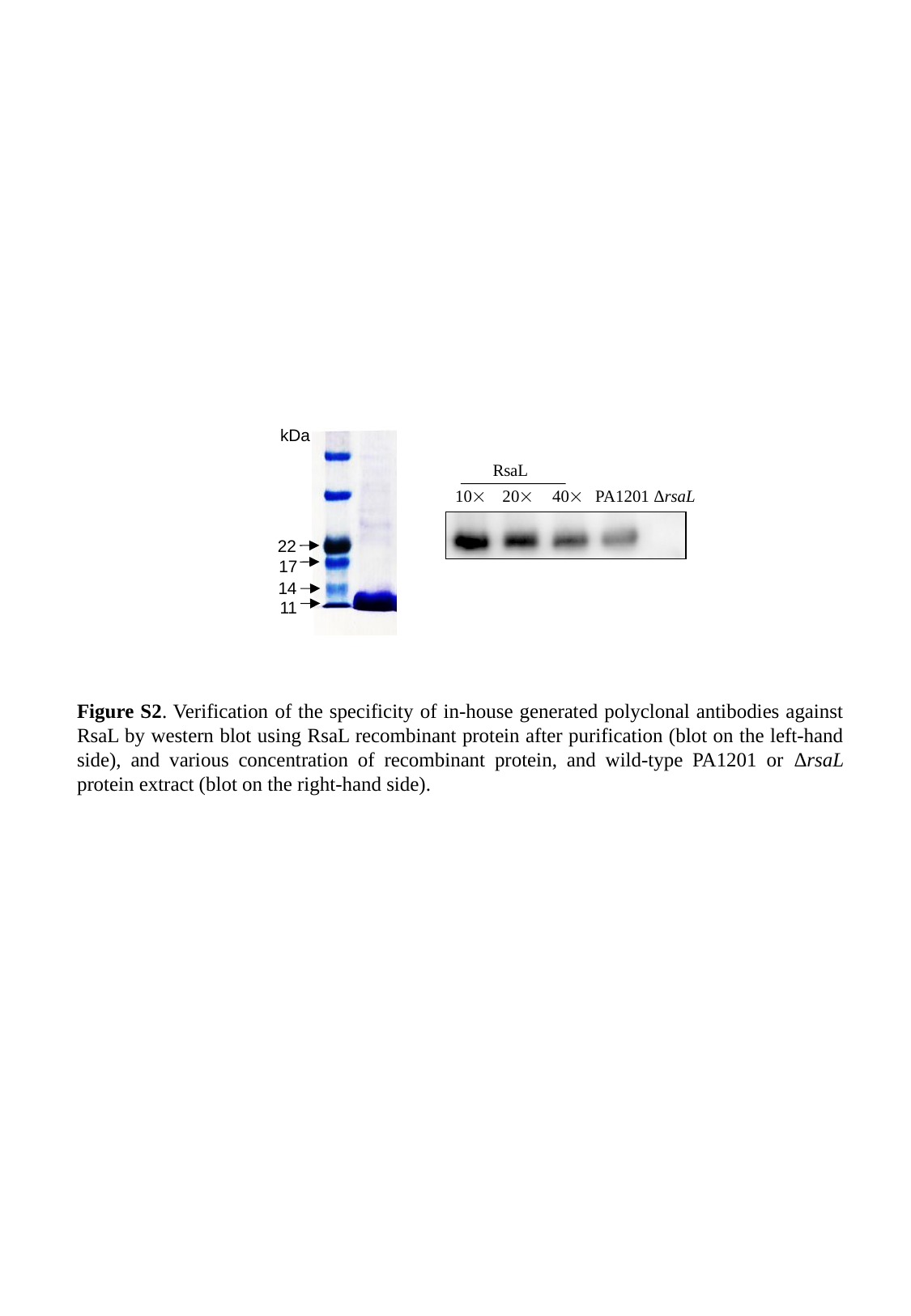

kDa
22
17
14
11
RsaL
10× 20× 40× PA1201 ΔrsaL
Figure S2. Verification of the specificity of in-house generated polyclonal antibodies against RsaL by western blot using RsaL recombinant protein after purification (blot on the left-hand side), and various concentration of recombinant protein, and wild-type PA1201 or ΔrsaL protein extract (blot on the right-hand side).

## Slide 3
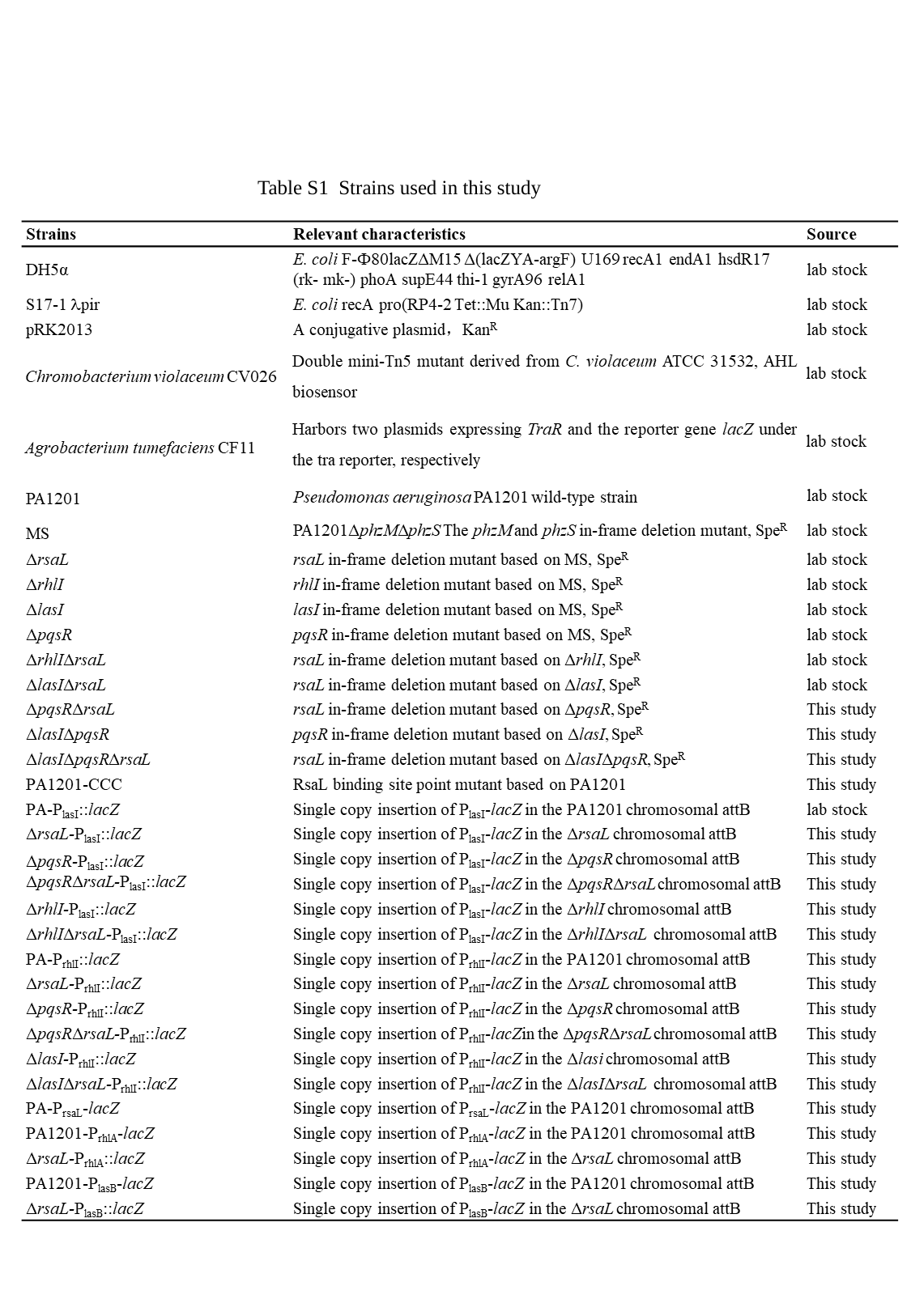

# Table S1 Strains used in this study

## Slide 4
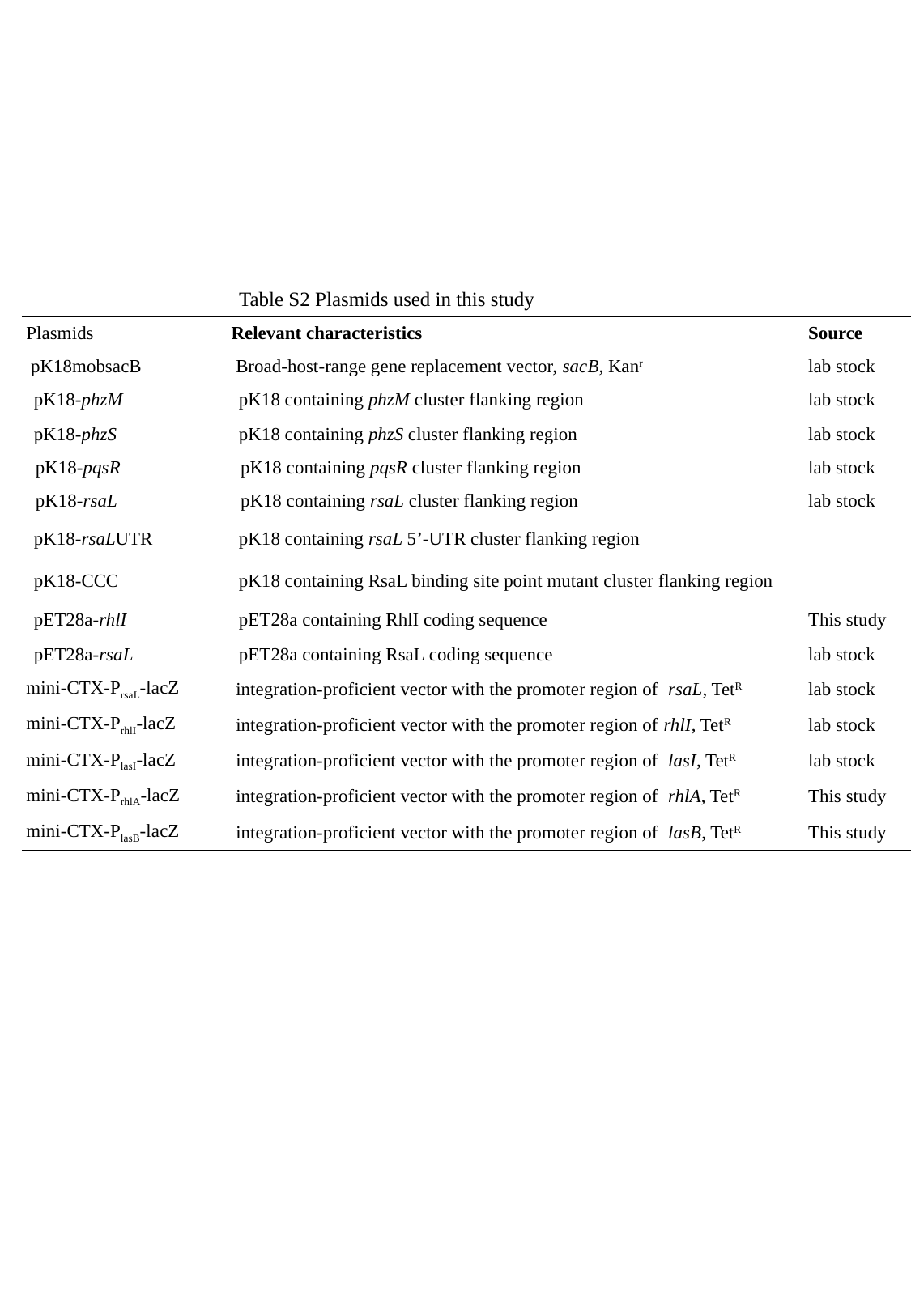

Table S2 Plasmids used in this study
| Plasmids | Relevant characteristics | Source |
| --- | --- | --- |
| pK18mobsacB | Broad-host-range gene replacement vector, sacB, Kanr | lab stock |
| pK18-phzM | pK18 containing phzM cluster flanking region | lab stock |
| pK18-phzS | pK18 containing phzS cluster flanking region | lab stock |
| pK18-pqsR | pK18 containing pqsR cluster flanking region | lab stock |
| pK18-rsaL | pK18 containing rsaL cluster flanking region | lab stock |
| pK18-rsaLUTR | pK18 containing rsaL 5’-UTR cluster flanking region | |
| pK18-CCC | pK18 containing RsaL binding site point mutant cluster flanking region | |
| pET28a-rhlI | pET28a containing RhlI coding sequence | This study |
| pET28a-rsaL | pET28a containing RsaL coding sequence | lab stock |
| mini-CTX-PrsaL-lacZ | integration-proficient vector with the promoter region of rsaL, TetR | lab stock |
| mini-CTX-PrhlI-lacZ | integration-proficient vector with the promoter region of rhlI, TetR | lab stock |
| mini-CTX-PlasI-lacZ | integration-proficient vector with the promoter region of lasI, TetR | lab stock |
| mini-CTX-PrhlA-lacZ | integration-proficient vector with the promoter region of rhlA, TetR | This study |
| mini-CTX-PlasB-lacZ | integration-proficient vector with the promoter region of lasB, TetR | This study |

## Slide 5
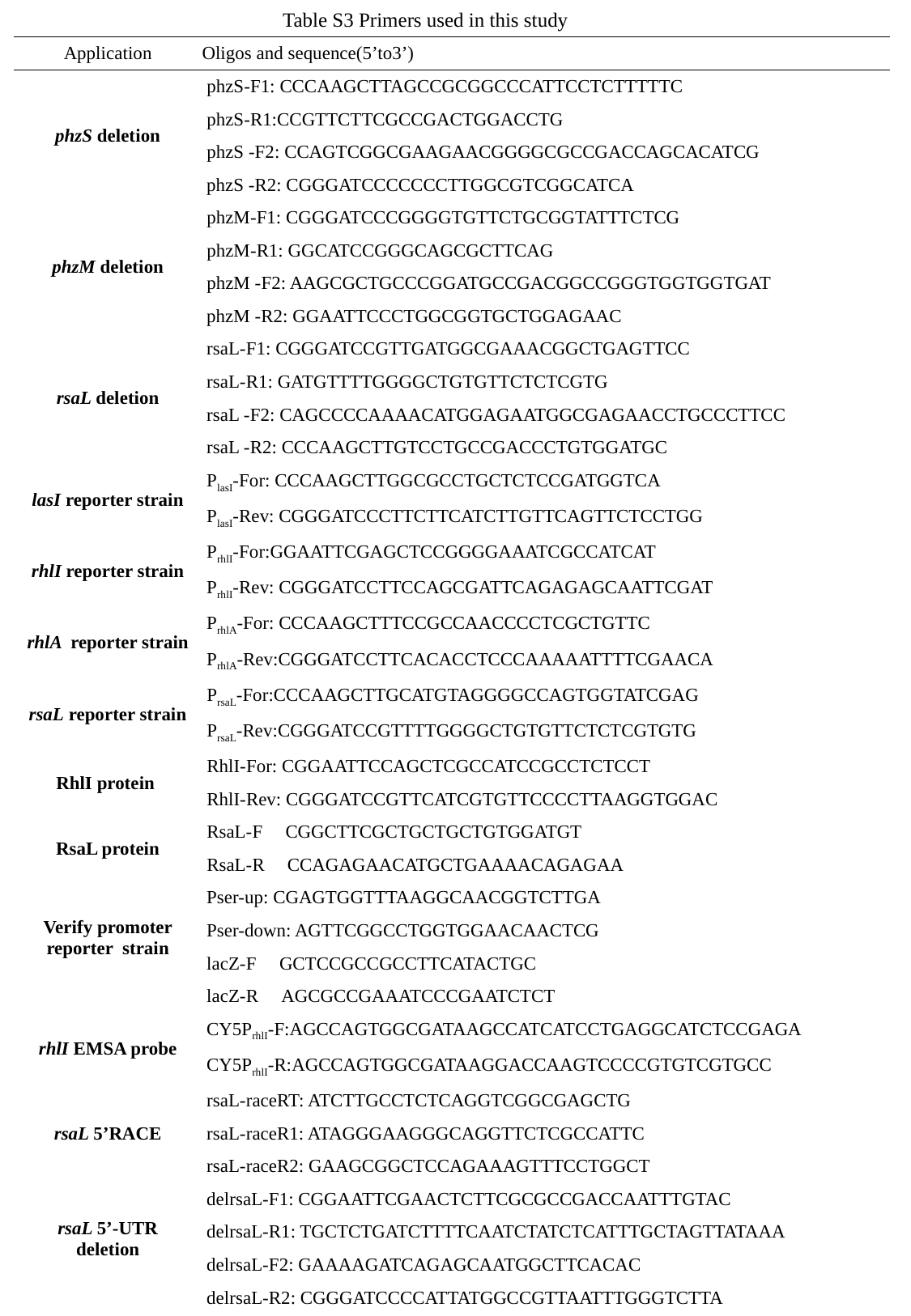

# Table S3 Primers used in this study
| Application | Oligos and sequence(5’to3’) |
| --- | --- |
| phzS deletion | phzS-F1: CCCAAGCTTAGCCGCGGCCCATTCCTCTTTTTC |
| | phzS-R1:CCGTTCTTCGCCGACTGGACCTG |
| | phzS -F2: CCAGTCGGCGAAGAACGGGGCGCCGACCAGCACATCG |
| | phzS -R2: CGGGATCCCCCCCTTGGCGTCGGCATCA |
| phzM deletion | phzM-F1: CGGGATCCCGGGGTGTTCTGCGGTATTTCTCG |
| | phzM-R1: GGCATCCGGGCAGCGCTTCAG |
| | phzM -F2: AAGCGCTGCCCGGATGCCGACGGCCGGGTGGTGGTGAT |
| | phzM -R2: GGAATTCCCTGGCGGTGCTGGAGAAC |
| rsaL deletion | rsaL-F1: CGGGATCCGTTGATGGCGAAACGGCTGAGTTCC |
| | rsaL-R1: GATGTTTTGGGGCTGTGTTCTCTCGTG |
| | rsaL -F2: CAGCCCCAAAACATGGAGAATGGCGAGAACCTGCCCTTCC |
| | rsaL -R2: CCCAAGCTTGTCCTGCCGACCCTGTGGATGC |
| lasI reporter strain | PlasI-For: CCCAAGCTTGGCGCCTGCTCTCCGATGGTCA |
| | PlasI-Rev: CGGGATCCCTTCTTCATCTTGTTCAGTTCTCCTGG |
| rhlI reporter strain | PrhlI-For:GGAATTCGAGCTCCGGGGAAATCGCCATCAT |
| | PrhlI-Rev: CGGGATCCTTCCAGCGATTCAGAGAGCAATTCGAT |
| rhlA reporter strain | PrhlA-For: CCCAAGCTTTCCGCCAACCCCTCGCTGTTC |
| | PrhlA-Rev:CGGGATCCTTCACACCTCCCAAAAATTTTCGAACA |
| rsaL reporter strain | PrsaL-For:CCCAAGCTTGCATGTAGGGGCCAGTGGTATCGAG |
| | PrsaL-Rev:CGGGATCCGTTTTGGGGCTGTGTTCTCTCGTGTG |
| RhlI protein | RhlI-For: CGGAATTCCAGCTCGCCATCCGCCTCTCCT |
| | RhlI-Rev: CGGGATCCGTTCATCGTGTTCCCCTTAAGGTGGAC |
| RsaL protein | RsaL-F：CGGCTTCGCTGCTGCTGTGGATGT |
| | RsaL-R：CCAGAGAACATGCTGAAAACAGAGAA |
| Verify promoter reporter strain | Pser-up: CGAGTGGTTTAAGGCAACGGTCTTGA |
| | Pser-down: AGTTCGGCCTGGTGGAACAACTCG |
| | lacZ-F：GCTCCGCCGCCTTCATACTGC |
| | lacZ-R：AGCGCCGAAATCCCGAATCTCT |
| rhlI EMSA probe | CY5PrhlI-F:AGCCAGTGGCGATAAGCCATCATCCTGAGGCATCTCCGAGA |
| | CY5PrhlI-R:AGCCAGTGGCGATAAGGACCAAGTCCCCGTGTCGTGCC |
| rsaL 5’RACE | rsaL-raceRT: ATCTTGCCTCTCAGGTCGGCGAGCTG |
| | rsaL-raceR1: ATAGGGAAGGGCAGGTTCTCGCCATTC |
| | rsaL-raceR2: GAAGCGGCTCCAGAAAGTTTCCTGGCT |
| rsaL 5’-UTR deletion | delrsaL-F1: CGGAATTCGAACTCTTCGCGCCGACCAATTTGTAC |
| | delrsaL-R1: TGCTCTGATCTTTTCAATCTATCTCATTTGCTAGTTATAAA |
| | delrsaL-F2: GAAAAGATCAGAGCAATGGCTTCACAC |
| | delrsaL-R2: CGGGATCCCCATTATGGCCGTTAATTTGGGTCTTA |
| rhlI RsaL binding site point mutant | Porb-F: TCATGTGTGTGCTGGCCCGTCCTCCGACTGAGA |
| | Porb-R: TCTCAGTCGGAGGACGGGCCAGCACACACATGA |
| rsaL EMSA probe | ErsaL-HF: AGCCAGTGGCGATAAGGAGATAGATTTCGGTGAACCCGGACCC |
| | ErsaL-HR: AGCCAGTGGCGATAAGGCTTATCCCGAAGCGGCTCCAGAAA |
| | ErsaLQF: AGCCAGTGGCGATAAGGCGTCATAACCATCGATTTCCATCTC |
| | ErsaL-QR: AGCCAGTGGCGATAAGGCTTCCTATTTGGAGGAAGTGAAGATGA |
| | EBS12-F: AGCCAGTGGCGATAAGATGCAAATTTCATAATTTTATAACTAGCA |
| | EBS12-R: AGCCAGTGGCGATAAGGTCCGGGTTCACCGAAATCTATCTCAT |
